# Genome and proteome of the chlorophyll *f*-producing cyanobacterium *Halomicronema hongdechloris*: adaptative proteomic shifts under different light conditions

**DOI:** 10.1186/s12864-019-5587-3

**Published:** 2019-03-12

**Authors:** Min Chen, Miguel A. Hernandez-Prieto, Patrick C. Loughlin, Yaqiong Li, Robert D. Willows

**Affiliations:** 1 School of Life and Environmental Sciences University of Sydney, Sydney, NSW 2006 Australia; 2 Department of Molecular Sciences Macquarie University, Sydney, NSW 2109 Australia

**Keywords:** Photosynthetic adaptation, Quantitative proteomics, Far-red light adaptation, *Halomicronema hongdechloris*

## Abstract

**Background:**

*Halomicronema hongdechloris* was the first cyanobacterium to be identified that produces chlorophyll (Chl) *f*. It contains Chl *a* and uses phycobiliproteins as its major light-harvesting components under white light conditions. However, under far-red light conditions *H. hongdechloris* produces Chl *f* and red-shifted phycobiliprotein complexes to absorb and use far-red light. In this study, we report the genomic sequence of *H. hongdechloris* and use quantitative proteomic approaches to confirm the deduced metabolic pathways as well as metabolic and photosynthetic changes in response to different photo-autotrophic conditions.

**Results:**

The whole genome of *H. hongdechloris* was sequenced using three different technologies and assembled into a single circular scaffold with a genome size of 5,577,845 bp. The assembled genome has 54.6% GC content and encodes 5273 proteins covering 83.5% of the DNA sequence. Using Tandem Mass Tag labelling, the total proteome of *H. hongdechloris* grown under different light conditions was analyzed. A total of 1816 proteins were identified, with photosynthetic proteins accounting for 24% of the total mass spectral readings, of which 35% are phycobiliproteins. The proteomic data showed that essential cellular metabolic reactions remain unchanged under shifted light conditions. The largest differences in protein content between white and far-red light conditions reflect the changes to photosynthetic complexes, shifting from a standard phycobilisome and Chl *a-*based light harvesting system under white light, to modified, red-shifted phycobilisomes and Chl *f-*containing photosystems under far-red light conditions.

**Conclusion:**

We demonstrate that essential cellular metabolic reactions under different light conditions remain constant, including most of the enzymes in chlorophyll biosynthesis and photosynthetic carbon fixation. The changed light conditions cause significant changes in the make-up of photosynthetic protein complexes to improve photosynthetic light capture and reaction efficiencies. The integration of the global proteome with the genome sequence highlights that cyanobacterial adaptation strategies are focused on optimizing light capture and utilization, with minimal changes in other metabolic pathways. Our quantitative proteomic approach has enabled a deeper understanding of both the stability and the flexibility of cellular metabolic networks of *H. hongdechloris* in response to changes in its environment.

**Electronic supplementary material:**

The online version of this article (10.1186/s12864-019-5587-3) contains supplementary material, which is available to authorized users.

## Background

Cyanobacteria were largely responsible for the initial oxygenation of Earth’s atmosphere approximately 3.6 billion years ago, performing oxygenic photosynthesis similar to plants [1]. Cyanobacteria are primary producers, widely distributed in seawater and fresh water systems, as well as terrestrial systems, thriving in extreme environments characterized by temperature stress, water limitation or light limitation [2–4]. Cyanobacteria have been and continue to be central to life on Earth. Genomic information available on cyanobacteria is rapidly accumulating due to high-throughput genome sequencing initiatives over the past three decades. To date, there are more than 1000 cyanobacterial genomic drafts deposited in public databases, including NCBI, CyanoBase, JGI and IMG/M (Integrated Microbial Genomes and Microbiomes).

Chlorophylls (Chls) are essential for oxygenic photosynthesis, however their absorbance characteristics can limit photosynthetic efficiency [5]. Chl absorbance maxima are at both extremes of the visible spectrum. The most prevalent Chls found in nature, Chl *a* and Chl *b*, cannot efficiently use light of wavelengths greater than 700 nm [6]. Chl *d* and Chl *f* can use light of wavelengths greater than 700 nm efficiently because of their red-shifted absorption properties, and as such they are called “red-shifted chlorophylls” [5–8]. These red-shifted Chls have been found in cyanobacteria that can thrive in extreme low light conditions, especially in light filtered by Chl *a/b*-containing organisms, where visible light is scarce and far-red (FR) light (> 700 nm), is more available [9–14].

Adaptation to the use of FR light for photochemical reactions requires remodelling of the photosynthetic machinery and their pigment composition [9–11, 15–18]. Since Chl *f* was first reported in 2010, isolated from the cyanobacterium *Halomicronema hongdechloris* [19], a number of species have been identified that accumulate Chl *f* and trace amounts of Chl *d* when grown under FR light [11, 13, 20, 21]. In these characterized Chl *f*-producing cyanobacteria, a gene cluster induced under FR light, the FaRLiP (far-red light photoacclimation) gene cluster, has been identified [21]. The FaRLiP cluster includes genes encoding subunits of photosystem I (PSI; *psa* genes) and photosystem II (PSII; *psb* genes) as well as genes encoding allophycocyanin proteins (*apc*) and regulatory proteins (*rfp*).

The PSII reaction center D1 protein is encoded by a small family of *psbA* genes. Most cyanobacteria possess multiple copies of *psbA* homologs, which are differentially regulated and used under different stress conditions [22]. Recently, cloning and expression in *Synechococcus* sp. PCC 7002 of a super rogue-*psbA* gene (*psbA4*) coming from the FaRLiP cluster of *Chlorogloepsis fritschii* PCC 9212 was shown to oxidize Chl *a* and to produce Chl *f* under illumination*.* This gene has since been reannotated as encoding a light-dependent chlorophyll *f* synthase and named *chlF* [23].

*H. hongdechloris* is a filamentous cyanobacterium originally isolated from a stromatolite in the World Heritage site of Shark Bay, Western Australia [9]. Under white light (WL) culture conditions it contains Chl *a* as its major photopigment and uses phycobilisomes as the dominant antenna system. However, under FR low light conditions *H. hongdechloris* uses both Chl *a* and Chl *f* for photosynthesis and has a higher growth rate than that under WL low light conditions [24]. *H. hongdechloris* was the first identified Chl *f*-producing cyanobacterium with a light quality-dependent switchable photosynthetic apparatus [9, 17, 25, 26]. Chl *f* has been detected in isolated PSI and PSII under FR light conditions [25, 27] and it has been postulated that uphill energy transfer mechanisms may occur in photosynthetic systems containing Chl *a* and Chl *f* [28–30]. In addition to producing Chl *f* under FR light conditions, *H. hongdechloris* restructures its phycobiliprotein complexes by significantly decreasing the expression of some subunits and incorporating newly synthesized phycobiliproteins that have a red-shifted absorption [9, 15, 17, 26]. Using ^18^O labelling technology we traced the Chl *f* biosynthetic pathway and confirmed that Chl *f* is synthesized from Chl *a* and requires the presence of oxygen molecules [31]. Chl *f* is degraded to undetectable levels within a week upon a shift to WL conditions [24, 31]. Despite this progress in our understanding, open questions remain about the biosynthesis and degradation of Chl *f*, as well as changes in photosynthetic apparatus, when shifting from FR to WL conditions, and vice versa.

In this study, we have sequenced the genome of *H. hongdechloris* and determined its genomic features. Making use of the annotated gene sequences, we combined absolute and relative whole-proteome quantitative analyses [32] to measure changes in *H. hongdechloris* cultures under two different light conditions at the protein level. These data reveal a number of coordinated changes within protein clusters and pathways in response to changes in light conditions.

## Results

### Genome sequence assembly

**Fig. 1:**
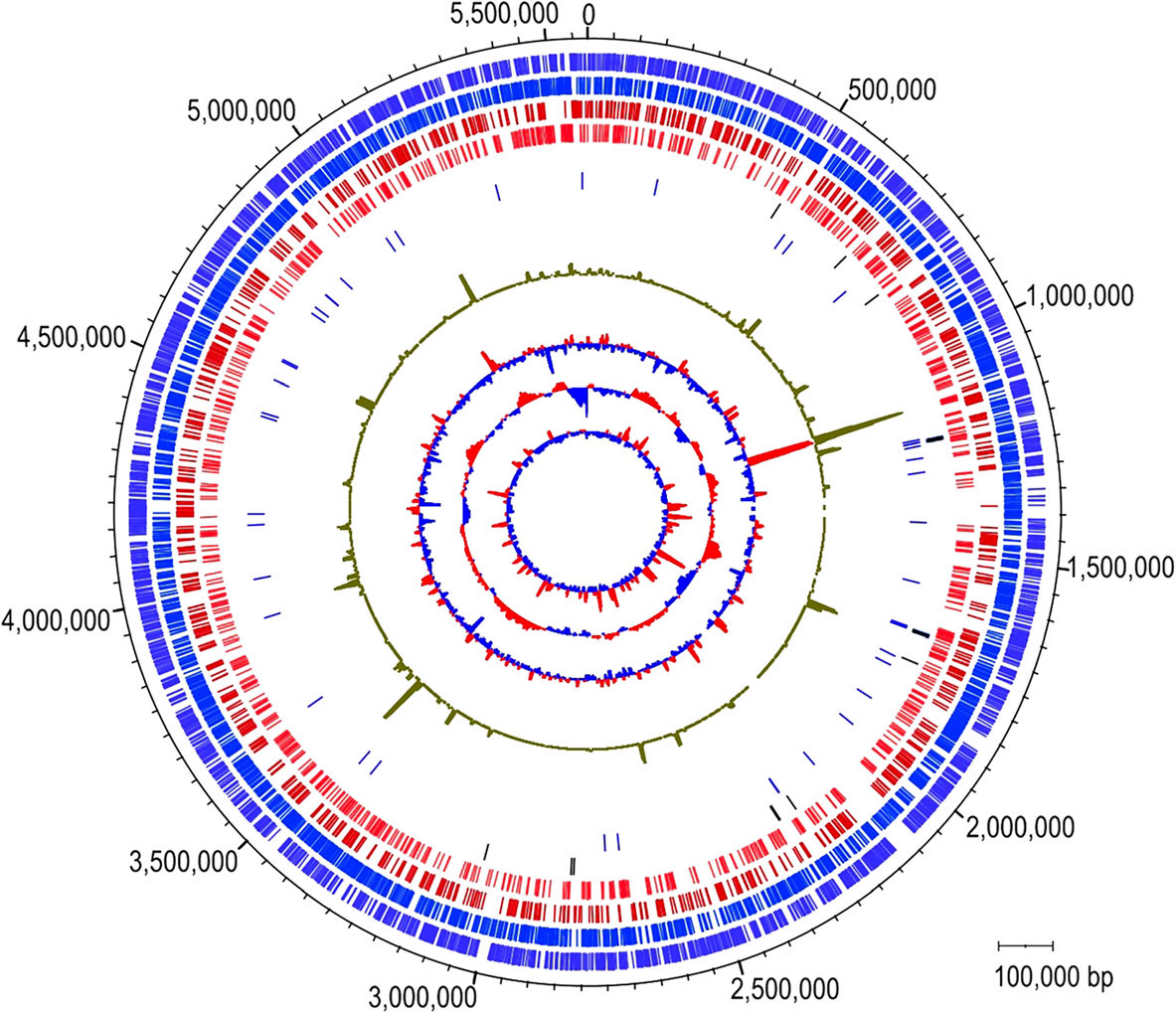
Genomic map of *H. hongdechloris* (bp). The rings from outer to inner represent: 1, genes on + strand (purple-blue color); 2, genes on – strand (blue); 3, MSMS detected proteins on + strand (red); 4, MSMS detected proteins on – strand (red); 5, rRNAs (blue) and CRISPR repeats (black); 6, tRNAs and tmRNA (blue); 7, Protein expression from spectral counts (green); 8, Differential protein expression in far-red light cells (red) compared to white light-grown cells (grey blue); 9, GC-skew; 10, sequence coverage (maximum 179x). Scale bar represents 100,000 bp

The *H. hongdechloris* DNA sequence reads were assembled de novo in a hybrid assembly using MIRA 4.9.2 [33] with further analysis using MUMmer [34] and manual contig joining in Gap5 [35] using PacBio long reads as described in the methods. The assembly of 19 contigs had N50 = 621,104 nt and N90 = 236,494 nt and was calculated to have an average coverage of 72x over all contigs with 17 × 454, 14x PacBio, and 41.5x Solexa sequence coverage. Further Sanger sequencing of PCR products and scaffolding joined these 19 contigs into a single circular scaffold with a genome size of 5,577,845 bp (Fig. 1).

### Genome annotation and overview

The single circular *H. hongdechloris* genome has a 54.62% GC content. The genome was annotated with BASYS [36], RAST [37], and PROKKA [38], and then the annotations manually compared and corrected in both Web Apollo [39] and Artemis [40]. Based on the three independent sequencing methods, we achieved an average of >72x genome coverage.

No plasmid was found in the genome of *H. hongdechloris.* The origin of replication is located at 5122382–5122353 nt with a single nucleotide difference between this and the *E. coli* perfect DnaA box (TTTTCCACA vs. TTTTCCACA) and the *oriC* region is next to a *dnaA* gene [41, 42]. There are no *terA*, *terB* or *terC-*like sequences, nor any *tus-*like genes that may be involved in termination of replication. There are two XerD site-specific recombinases, XM38_013470 and XM38_012510, which are known to be involved in termination of replication and resolving chromosome cocatamers together with Ftsk, XM38_048340 [43]. However, the *dif*/XerCD system of dimer resolution requires a *dif* site [43], and there are no predicted dif sites in *H. hongdechloris* or in other cyanobacteria so the role of these XerD recombinases in replication remains uncertain.

**Fig. 2:**
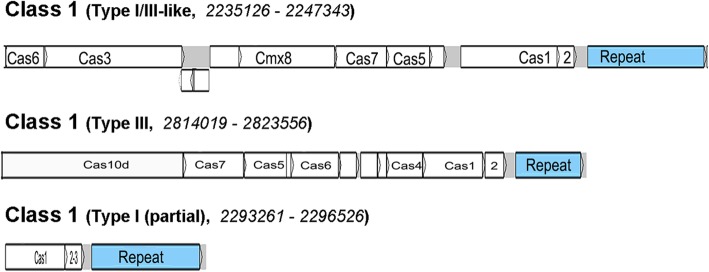
The organization of examples of *cas* operons from the *H. hongdechloris* genome. The definition of CRISPR-Cas types is as described in Westra et al. 2016 [44]. In addition to the examples presented here, two additional simplified Type I operons (*cas1* + *cas2–3*) are located in the genomic regions of 499,628–501,870, and 824,592–825,871, respectively

The *H. hongdechloris* genome contains 5335 genes with 5273 putative protein encoding genes (99.0%) (Table 1). Approximately 44% of the predicted protein coding genes are annotated as “hypothetical protein/conserved domain”. There is a single transfer messenger RNA (tmRNA), 45 tRNA genes, and two copies each of the 23S, 16S, and 5S rRNA genes (Table 1). There are multiple predicted CRISPR repeat operons and insertion arrays, including both Type I and Type III CRISPRs (Table 1). Three class 1 CRISPR-associated (*ca*s) operons were annotated (Fig. 2) [44]. The simplified type I operon organisation contains only *cas1* + *cas 2–3*, which represents the dominant operon organisation in *H. hongdechloris*. Five *cas1* and four *cas2–3* genes were annotated in the *H. hongdechloris* genome. There are 722 tandem repeats in the genome with the sizes in the range of 7 to 644 bp (Additional file 1: Excel file S1). The median size of the repeats was 14 bp with, on average, 4 repeats in tandem [45]. Using ISSaga (http://issaga.biotoul.fr/ISsaga2,), 123 ORFs were identified as putative insertion sequence (IS) elements. There were 62 different types of IS elements belonging to 22 different families with the longest of each of these IS elements included in Additional file 1: Excel file S1. There is a section at 3260831–3267639 nt containing phage-like proteins. This region contains five genes including two ABC transporter genes, an ISL3-type transposase, a hypothetical phage protein, and a gp245-type phage protein [46]. There are verified frame shifts in the transposase and one of the ABC transporters, suggesting these genes are non-functional.

**Table 1:** Features of the *H. hongdechloris* genome

Attributes	value	Percentage (%)
Genome size (bp)	5,577,845	100
DNA coding base pairs	4,652,307	83.4
G + C content	54.6%	
Total annotated genes	5335	100
Protein encoding genes	5273	98.84
RNA genes	45 tRNAs; 6 rRNAs; 1 tmRNA	0.96
Protein-encoding genes with predicted functions	2576	48.3
Hypothetical protein/conserved domains	2349	44.0
Confirmed proteins by proteomic analysis	1816	34.0
CRISPR repeats	10	0.2

### Photosynthetic apparatus

**Fig. 3:**
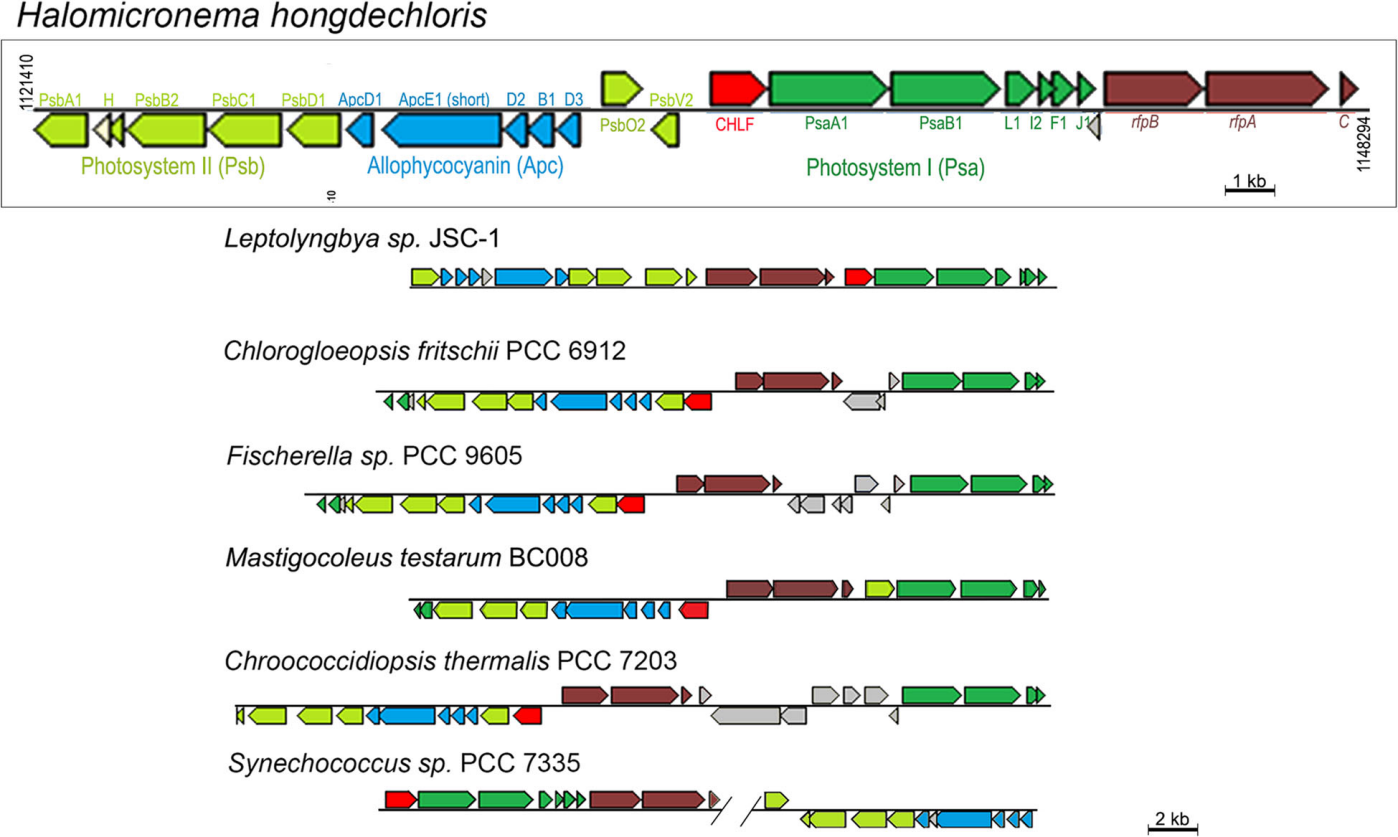
Comparison of the 21-gene FaRLiP gene cluster from *H. hongdechloris* with those from other cyanobacteria. The 21-gene FaRLiP gene cluster from *H. hongdechloris* includes genes encoding subunits for PSI (*Psa*s, in green) PSII (*Psb*s, in light green) and red-shifted allophycobiliprotein subunits (*Apc*s, in blue). The gene in red represents the Chl *f* synthase (ChlF) homolog, and Far-red light acclimation regulator (rfp) genes are in brown. Hypothetical proteins are shown in grey. Similar gene clusters from other cyanobacteria are compared with the same color codes. Scale bar for *H. hongdechloris* is 1 kb and 2 kb for the other cyanobacteria

There are 20 genes annotated for photosystem I (PSI; *psa* genes), 33 genes annotated for photosystem II (PSII; *psb* genes), and 22 genes encoding components of photosynthetic antenna systems, including chlorophyll-binding light-harvesting proteins (IsiA/CP43’ family) and phycobiliprotein complexes (Additional file 2: Table S1). The multiple copies of genes are annotated using the homologous gene name and numbers following their order in the genome. The core subunits of PSI comprise two large polypeptides PsaA and PsaB encoded by genes *psaA* and *psaB* of which there are three pairs in the genome. Phylogenetic analysis reveals that the three copies of PsaA belong to different groups (Additional file 2: Figure S1), with PsaA1 showing sequence similarities with PsaA proteins from the group of cyanobacteria that have the FaRLiP (far-red light photoacclimation) gene cluster (Fig. 3). Additionally, there are three copies of *psaI* and *psaL*, and two copies of *psaF* and p*saJ*. Interestingly, a *psaA/B/L/I/F/J* cluster is located under the same operon as the red-shifted allophycocyanin subunits (*apc*) and a group of core PSII proteins (Fig. 3).

**Fig. 4:**
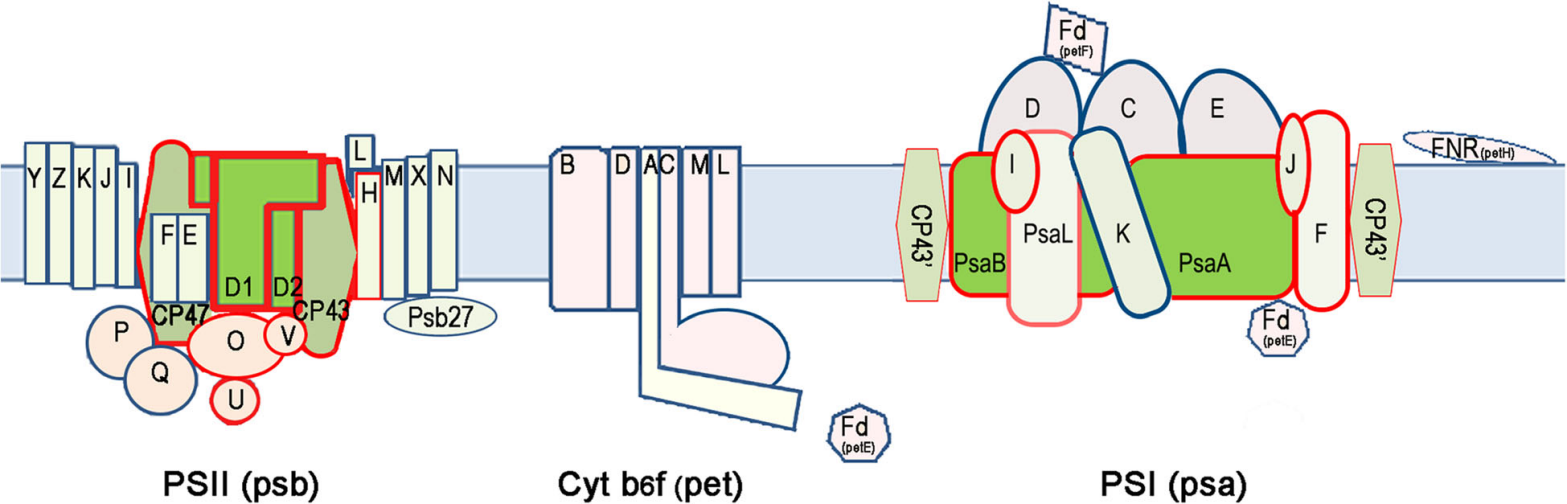
Schematic changes in photosynthetic complexes. Subunits that have a gene copy within the FaRLiP gene cluster are framed in red. The schematic model was drawn after cyanobacterial PSI (PDB 2001) and PSII (PDB 2AXT) crystal structures

Multiple copies of *psa/psb* genes encoding proteins associated with both photosystems were identified (Fig. 4). There are 5 copies of genes encoding PsbA homologs including *chlF*, XM38_010900, previously termed super rogue-*psbA* [47] named, which is proposed to encode the Chl *f* synthase [23]. These five PsbA homologs are phylogenetically grouped in three branches, including a ChlF synthase cluster (Additional file 2: Figure S2). In addition to the multiple copies of *psbA,* there are also two copies of genes encoding the PSII core subunits PsbB, PsbC, PsbH, PsbO, and PsbV (Fig. 4). No *psbT* homologs were found in the *H. hongdechloris* genome (Additional file 2: Table S1). PsbT is reported to play a role in stabilizing the structure of PSII under high-light stress conditions, as a null mutant of PsbT in *Synechocystis* PCC 6803 could grow under moderate light (40 μmol photons m^− 2^ s^− 1^) but not under high-light conditions (~ 4000 μmol photons m^− 2^ s^− 1^) [48]. Since *H. hongdechloris* is adapted to a low/filtered light environment, the apparent absence of PsbT is not unexpected.

The genomic sequence data provide evidence that *H. hongdechloris* can produce both chlorophyll-binding internal membrane antennae and peripheral external membrane phycobiliprotein antennae. Two chlorophyll-binding antenna homologs (XM38_005580 and XM38_020880) are annotated as *isiA* (Additional file 2: Table S1). These IsiA are predicted to contain 6 transmembrane domains and conserved chlorophyll binding sites similar to members of the CP43 family [49]. Interestingly, the IsiA2 encoded by XM38_020880 contains an extension of ~ 150 aa at the C-terminus (Additional file 2: Figure S3). This gene phylogenetically clustered with other IsiA-like homologs from Chl *f*-producing cyanobacteria, which also contain this extension (Additional file 2: Figure S3). This C-terminal extension contains numerous glutamine and proline residues and it would be expected to extend outside of the thylakoid membranes, however its function is unknown. Of note, two histidine residues, conserved in almost all reported CP43’/IsiA [49], are missing in the putative 6th helix region of all of the IsiA proteins that have this C-terminal extension.

Phycobiliproteins assemble to form phycobilisomes, which function as the major antenna system in *H. hongdechloris*, allowing it to take advantage of different light environments [17]. There are 20 genes annotated as phycobiliprotein complex-related, including 12 genes encoding allophycocyanin subunits (Additional file 2: Table S1C). *H. hongdechloris* contains only one copy of the *cpcA-cpcF* operon, which encodes the basic phycocyanin units in the peripheral phycobiliprotein antenna. Multiple copies of allophycocyanin subunits are present: one ApcA, four ApcB, five ApcD, and two ApcE-encoding genes. Of the two predicted ApcE proteins (XM38_010840 and XM38_033070), ApcE1 (XM38_010840, annotated ApcE1 following the gene order in the genome) contains two phycobilisome linker domains (Pfam00427), whereas ApcE2 (XM_033070) has four phycobilisome-binding Pfam00427 domains. The numbers of phycobilisome-binding domains determine the types of phycobilisomes produced. That is, two phycobilisome-binding domains will only support a small phycobilisome size, as observed under FR light conditions in a previous report [17]. Three out of the five ApcD subunits are located in the gene cluster under the control of FaRLiP regulators, together with the core subunits of PSI and PSII (Fig. 3). FaRLiP regulators consist of the phytochrome RfpA (XM38_010990) and a two-component regulatory system, RfpB (XM38_010980) and RfpC (XM38_011000).

### Energy metabolism

Carbohydrates are one of the major forms of energy storage for living organisms. The energy stored in carbohydrates is released in the form of ATP and NAD(P)H in a set of reactions that feed reduced substrates to the oxidative phosphorylation pathway. The *H. hongdechloris* genome contains genes encoding the four complexes responsible for the electron transfer chain within oxidative phosphorylation: NAD(P)H dehydrogenase complex (Complex I), succinate dehydrogenase (Complex II), cytochrome *c* oxidase (Complex IV), and ATPase (Complex V) (Additional file 2: Table S2).

Photoautotrophic cyanobacteria can synthesize carbohydrates from CO_2_ via the Calvin-Benson cycle. Genes encoding the enzymes of the Calvin-Benson cycle were identified in *H. hongdechloris*, and are similar to those found in other cyanobacteria (Additional file 2: Figure S4). In cyanobacteria, glucose can be catabolized through three common glycolytic pathways, glycolysis (Embden-Meyerhof-Parnas pathway, EMP), the oxidative pentose phosphate pathway (OPP), and the Entner-Doudoroff pathway (ED). We identified the majority of genes encoding enzymes in these pathways within the genome of *H. hongdechloris* (Additional file 2: Figure S4). The OPP can run in either direction, to oxidise carbohydrates, or in its reductive mode to provide intermediates to the Calvin-Benson cycle to fix CO_2_. Interestingly, the *H. hongdechloris* genomic annotation showed that it may lack a typical cyanobacterial fumarate hydratase (fumarase) (EC 4.2.1.2), which converts fumarate to malate (Additional file 2: Figure S5). An annotated argininosuccinate lyase (XM38_040900) showed > 78% amino acid identity with annotated cyanobacterial argininosuccinate lyases (data not shown). This lyase belongs to the lyase-I-like superfamily (pfam00206) which also includes fumarate hydratase (Accession: cl00013). Whether or not XM38_040900 can catalyze the synthesis of malate from fumarate, and complete the TCA cycle in *H. hongdechloris*, requires further experimental confirmation. The TCA cycle and associated metabolic pathways (Additional file 2: Figure S5) may also play ancillary roles in biosynthesis in addition to its primary role in energy metabolism.

The genomic information showed that *H. hongdechloris* has the potential to synthesize different types of polysaccharides and sugars, such as glycogen/starch, cellulose and the disaccharide trehalose (Additional file 2: Figure S6). *H. hongdechloris* may use glycogen/starch as a secondary short-term energy store and break it down to glucose (Additional file 2: Figure S6). To examine the ability of *H. hongdechloris* to utilize environmental organic carbon compounds, we measured the growth rate of cultures supplemented with various additives. Cultures with either supplementary 0.1% soluble starch or 0.1% mannitol had enhanced growth rates (Additional file 2: Figure S6B), suggesting *H. hongdechloris* may be able to utilize these compounds as a carbon source. We also identified two enzymes predicted to catalyze the synthesis and degradation of trehalose (XM38_0499940 and XM38_049950), which are not commonly present in cyanobacteria. As *H. hongdechloris* was isolated from stromatolites [9], which are in intertidal regions subject to dehydration at low tide, trehalose is a likely contributor to the ability of *H. hongdechloris* to survive these transient dehydration conditions.

Nucleotide sugars act as donors of sugar residues in glycosylation reactions that produce polysaccharides. They are also the intermediates in nucleotide sugar interconversions whereby activated sugars are synthesized to meet the needs of glycosylation reactions (Additional file 2: Figure S6A). The presence of two copies of ADP-glucose synthases (XM38_014150 and XM38_031230) support the importance of biosynthesis of nucleotide sugars in *H. hongdechloris*.

### Chlorophyll biosynthesis

**Fig. 5:**
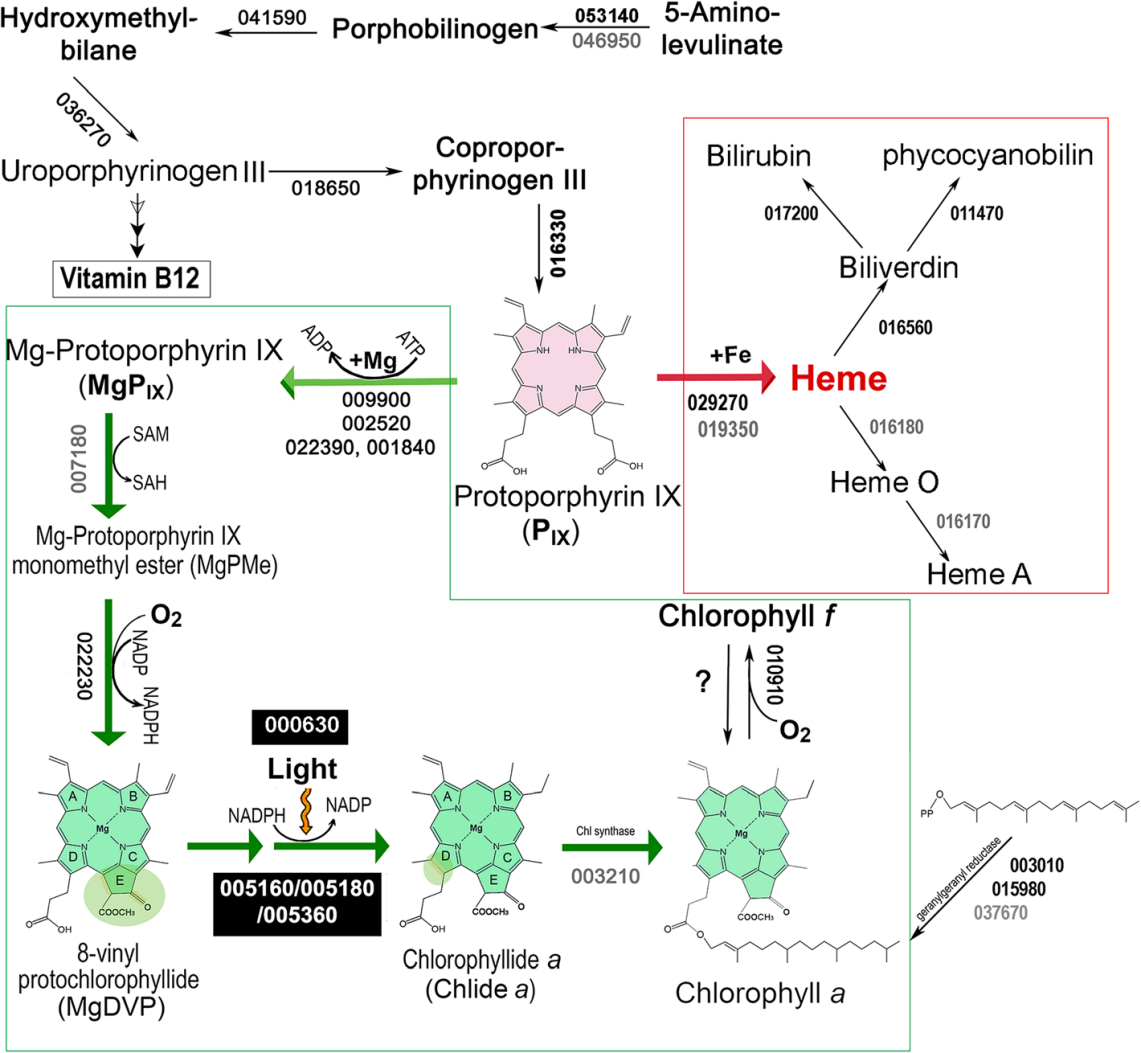
Chlorophyll and heme biosynthetic pathways with annotated genes from the *H. hongdechloris* genome. The enzymes in white type on a black background showed changes in abundance in response to changed light conditions. Proteins encoded by grey colored genes were below the detection limits of our proteomic study

All genes needed for Chl *a* biosynthesis have been identified in the genome and are distributed throughout the genome (Fig. 5). Most genes involved in Chl *a* biosynthesis are present as single copies except protoporphyrin IX Mg-chelatase H subunit (*chlH*) and geranylgeranyl reductase (*chlP*)(Fig. 5). There are two copies of *chlH* (*XM38_001840* and *XM38_022390*) and three copies of *chlP* (*XM38_003010, XM38_015980* and *XM38_037670*). As noted above, the PsbA homolog XM38_010900 is believed to encode Chl *f* synthase, although the degradation pathway of Chl *f* remains unknown.

### Proteomic analysis

We compared the proteome of *H. hongdechloris* cells grown under FR light with those grown under WL conditions, as well as cells that were switched from one light condition to the other, using Tandem Mass Tag (TMT) labelling technology. A total of 1816 proteins were detected at a false discovery rate of 0.5%, which accounted for 34.4% of predicted encoded proteins. Of the 1816 proteins detected, 574 were proteins of uncharacterized function. Of the remaining 1242 proteins, 960 had either an assigned GO-biological process category, an EC number, or a predicted enzymatic or binding activity. These functions covered all of the central metabolic pathways expected for autotrophic organisms (Additional file 2: Figure S7A). Photosynthesis-related proteins (including light-harvesting proteins and photosynthetic pigment-protein complexes) represent 24% of the detected proteins. Nearly 5% of the detected proteins are involved in glycolysis/gluconeogenesis, 3% involved in photosynthetic carbon fixation and only about 5.5% of these 1242 proteins had no functional annotation (Additional file 2: Figure S7A).

Using mass based spectral abundance factor (SAF) assessment, we determined the 100 most abundant proteins, which individually account for between 0.20 and 2.10% of total protein (Additional file 3: Excel file S2). Three abundant protein clusters (highlighted in Additional file 2: Figure S8) correspond to phycobiliprotein subunits. The most abundant protein, accounting for 2.1% of total protein, is phycocyanin subunit A (CpcA) and is encoded by *XM38_016410* (Additional file 2: Figure S7; Additional file 3: Excel file S2). However, CpcA was significantly less abundant in FR light cells compared with WL cells (Additional file 3: Excel file 2). The top 20 most abundant proteins are mainly from the “energy production and conversion (C)” functional category and 14 out of these 20 proteins showed significant changes under changed light conditions (Table 2). The three most abundant proteins from the "translational ribosomal structure and biogenesis (J)" functional category showed no significant change in abundance under different light conditions (Table 2).

**Table 2:** Top 20 abundant proteins in cultured *H. hongdechloris* detected using TMT methods

Gene id and Protein names	Gene names	GO groups	NSAF
XM38_016410 C-phycocyanin alpha chain	*cpcA*	C	2.102094144
XM38_010860 Allophycocyanin beta chain	*apcB*	C	1.776656472
XM38_010840 Phycobiliprotein ApcE	*apcE1*	C	1.680191234
XM38_010810 Photosystem II CP43 reaction center protein	*psbC*	C	1.620297982
XM38_033090 Allophycocyanin beta chain	*apcB*	C	1.578160694
XM38_016420 Phycocyanin-Beta subunit	*cpcB*	C	1.512437125
XM38_048950 hypothetical protein (no response to light changes)		N/A	1.422597247
XM38_043620 ATP synthase subunit beta	*atpD*	C	1.371714484
XM38_011320 Ribulose bisphosphate carboxylase large chain	*rcaL*	C/G	1.309966131
XM38_006390 60 kDa chaperonin (*groL1*)	*groL1*	J	1.300160599
XM38_033070 Phycobiliprotein ApcE	*apcE2*	C	1.27127403
XM38_010880 Photosystem II manganese-stabilizing polypeptide	*psbO*	C	1.159967986
XM38_024350 60 kDa chaperonin (*groL2*)	*groL2*	J	1.089474158
XM38_033080 Allophycocyanin alpha subunit	*apcA*	C	1.071718194
XM38_016390 Phycobilisome 32.1 kDa linker polypeptide, phycocyanin-associated, rod	*cpcC*	C	1.024015604
XM38_009060 Elongation factor Tu	*tuf*	J	1.020570417
XM38_043770 Chaperone protein dnaK2	*dnaK2*	K	0.996454107
XM38_010800 Photosystem II CP47 reaction center protein	*psbB*	C	0.990623791
XM38_052610 Aliphatic amidase expression-regulating protein	*amiC*	N/A	0.902904028
XM38_031350 Transketolase	*tkt2*	C/G	0.858646625

C = energy production and conversion; G = carbohydrate transport and metabolism; J = translation, ribosomal structure and biogenesis; K = transcription; O = posttranslational modification, protein turnover, chaperones; N/A = not assigned

**Fig. 6:**
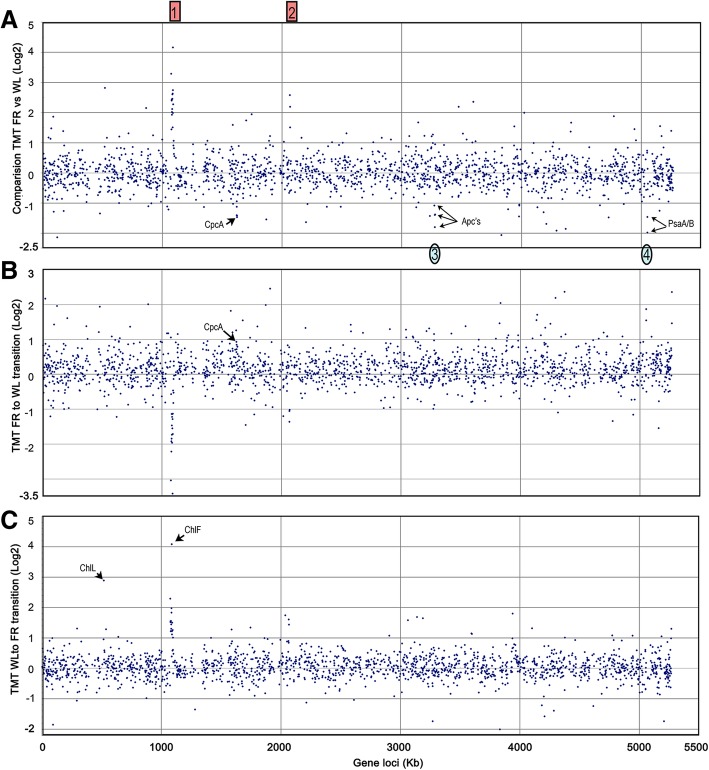
Genomic profiles of Log2 global protein abundance differences under differing light contains. Comparison of protein abundance in steady state FR light grown cells and WL grown cells (**a**). Protein abundance changes in response to shifting cells from FR light to WL conditions (**b**) or from WL to FR light conditions (**c**). The numbers in the red boxes highlight clusters of proteins that are stimulated by FR light conditions; the numbers in the blue ovals highlight clusters of proteins stimulated by WL conditions. Other proteins of interest are labelled

Figure 6a shows relative protein levels in FR-cells compared to mass readings from WL-cells. The proteomic changes when switching cells from FR to WL conditions (FRW-cells) were examined by comparing the mass readings from FRW-cells with the mass readings from FR-cells (Fig. 6b). Similarly, the dynamic proteomic changes when switching cells from WL to FR light conditions (WR-cells) were obtained by comparing mass readings from WR-cells with those from WL-cells (Fig. 6c). 118 out of 1815 detected proteins (7%) significantly changed in abundance (two-fold or greater increase or decrease) upon altered light conditions. 73 proteins showed significantly higher abundance in FR light cells and 45 proteins were significantly more abundant in cells grown under WL conditions (Fig. 6). Proteins from chlorophyll-binding complexes are the dominant group of proteins that increased in abundance in FR-cells, whereas phycobiliprotein subunits predominate in the proteins with higher abundance in WL cells (Additional file 2: Figure S7).

### Mechanisms that might be involved in responses to the switched light conditions

When light conditions were changed in either direction, the proteome of *H. hongdechloris* showed no significant quantitative changes in proteins essential for general cellular activities such as DNA/RNA metabolic reactions (repair, transcription and translation), protein synthesis and modifications, or carbohydrate-related energy metabolism (Additional file 3: Excel file S2). This implies that general cellular functions proceed as usual and that growth rate changes may simply be regulated by energy supply from the different types of photosynthetic complexes.

**Fig. 7:**
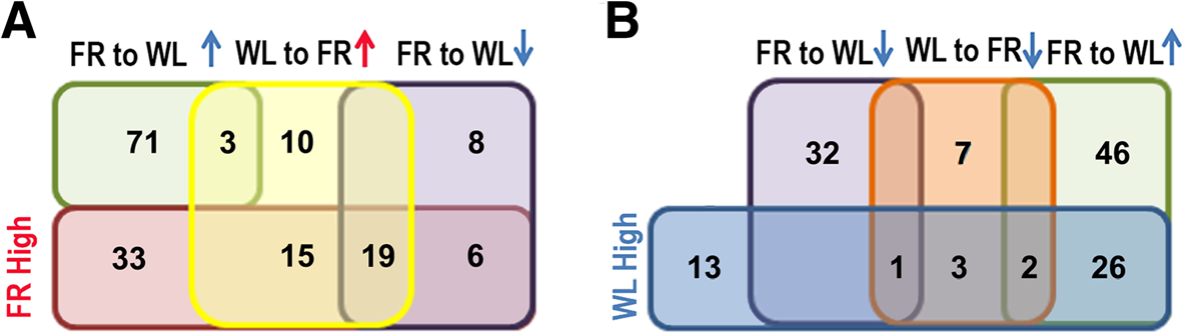
Venn diagram of proteins with changes in abundance under switched light conditions. “FR high” represents proteins more abundant in FR-cells (purple-red); “WL high” represents those more abundant in WL-cells (blue). FR to WL↑(or FR to WL↓) represents the upregulated (or downregulated) proteins when the cell culture is switched from FR light to WL light conditions (light green or purple color); WL to FR↑(or WL to FR↓) represents the upregulated (or downregulated) proteins when the cell culture is switched from WL light to FR light conditions (yellow or orange color). **a** Relationship of proteins with significantly increased abundance in FR-cells compared to WL-cells and those up and down-regulated in cells transferred from WL to FR light and vice versa respectively; **b** Relationship of proteins with significantly increased abundance in WL-cells compared to FR-cells and those up and down-regulated in cells transferred from FR to WL and vice versa respectively

Of the 73 significantly more abundant proteins observed in FR-cells, 19 proteins showed a ‘switchable’ response to changing light conditions (Fig. 7). That is, these 19 proteins were less abundant when FR-cells were switched to WL conditions (FRW-cells vs FR-cells), and more abundant in WL-cells that were switched to FR light conditions (WR-cells vs WL-cells). Of the 45 proteins that were more abundant in WL-cells, only three proteins demonstrated a similar ‘switchable’ response to changing light conditions (Fig. 7b). Of the 22 proteins with ‘switchable’ abundance, 16 are photosynthetic pigment-binding proteins, including *psa*’s, *psb*’s, and *apc*’s within the 21-gene FaRLiP cluster (Additional file 3: Excel file S2).

The abundance of 60 proteins changed significantly when the light was switched from WL to FR light, with 47 proteins upregulated by FR light condition and 13 proteins with a reduced abundance (Fig. 7b). The most upregulated protein, one week after WL-cells were switched to FR light conditions, was the Chl *f* synthase (ChlF, XM38_010900), with more than a 17-fold increase. The second most upregulated protein by FR light conditions, with a 7.5-fold increase, was the light-independent protochlorophyllide reductase subunit ChlL (XM38_005180; Fig. 5). Nineteen proteins showed significant counterpart changing profiles (i.e. up or down under switched FR light or WL conditions), 16 of which are photosynthetic pigment-binding proteins (Additional file 3: Excel file S2). Four proteins were significantly changed in response to switching the light conditions of the culture in either direction (FR to WL or vice versa). Three of these proteins are annotated as hypothetical proteins (XM38_044190 XM38_053170 and XM38_053180), and were consistently upregulated when switching light conditions. The fourth protein XM38_033020 (annotated as AtpH subunit), was down-regulated when switching light conditions, irrespective of the direction of light change (Additional file 3: Excel file S2).

The proteome profile of FR light-grown cells showed two clusters of proteins with a coordinated response to the light conditions (Fig. 6). Unsurprisingly, one of the clusters corresponds to the 21-gene FaRLiP cluster (XM38_010770 to XM38_010970), encoding subunits of photosynthetic complexes. This agrees well with previous reports, which have shown they are significantly upregulated under FR light [17, 25]. Another four-gene cluster (XM38_020870 to XM38_020900) containing genes encoding allophycocyanin subunits of *apcD* and *apcB*, PSII *psbA4* and *isiA2* genes also showed significant stimulation under FR light conditions. These two clusters demonstrate similar responses to the changed light conditions, however no far-red light acclimation regulator (*rfp*) genes were identified near the four-gene cluster. Apart from these two clusters, a pair of PsaA/B and allophycopbiliprotein subunits showed the opposite response, i.e. they are decreased under the FR light condition and increased in WL condition (Fig. 6).

## Discussion

We have sequenced and investigated the genome of *H. hongdechloris* and used proteomic analysis to examine relative and absolute protein abundance in cells grown under WL and FR light conditions. Furthermore, we have shown the dynamic changes in protein abundance when cells are shifted from WL to FR light conditions and vice versa. Our results support the notion that mechanisms for responding to FR light are conserved among Chl *f*-producing cyanobacteria, i.e. the abundance of proteins encoded by the 21-gene FaRLiP cluster is increased under FR light conditions [21]. However, there are subtle differences in this FaRLiP cluster amongst the known Chl *f*-producing cyanobacteria. For example, in *H. hongdechloris* there are additional copies of *psbO* and *psbV* genes (Fig. 3). These proteins increase under FR light conditions coordinately with the other photosynthetic proteins encoded by the genes in the FaRLiP cluster. However, the functions of these additional PsbO and PsbV proteins require further investigation. In addition, we have identified a four gene operon, *psbA*-*apcD*-*apcB*-*IsiA2*, that is also stimulated under FR light. The structure of this operon resembles that of the low light photoacclimation (LoLiP) cluster, of *apcD4*-*apcB3*-*isiX* identified in *Synechococcus* sp. [50]. It will be interesting to investigate the regulatory mechanism of this four-gene operon to determine whether its expression is also upregulated under low-light conditions or whether it represents a novel FR light response in *H. hongdechloris*. We have previously shown that subunits identified in the isolated red-shifted phycobilisomes are encoded in these two clusters and the annotated gene names have been corrected since completion of the genomic sequence following phylogenetic comparison [17]. ApcE1 and ApcE2 are annotated based on their genomic loci, independent of previous reported biochemical features. ApcE1 (previously ApcE2 [17]) contains two Pfam00427 domains and is involved in the construction of small red-shifted phycobilisomes, which is the dominant form of core-membrane linker protein in FR light cells [17]. Both *apcE* genes showed similar average abundance under WL and FR conditions, however, under FR light conditions ApcE1 (encoded by *XM38_010840*) is six-fold higher in abundance, and ApcE2 (encoded by *XM38_033070*) is three-fold lower. The proteomic profiles of phycobilisome subunits agree well with the previous report that smaller, allophycocyanin-containing phycobilisomes with red-shifted absorbance spectra are more abundant under FR light [17]. The five allophycocyanin ApcD subunits are also annotated based on their order in the genomic sequence with ApcD3 (encoded by *XM38_010870*) and ApcD4 (encoded by *XM38_020900*) replacing the previously named ApcA2 and ApcA3, which were misnamed due to an incomplete genome sequence [17].

There are multiple copies of subunits that make up the reaction centres of PSI and PSII. and they all have a copy in the FaRLiP cluster, suggesting they are required for adaptation to FR light conditions (Fig. 4). The PSI core proteins encoded by the three copies of *psaA* and *psaB* account for an average of ~ 1.0% of total protein. The PsaA1 and PsaB1 pair and PsaA3 and PsaB3 pair are the dominant components of PSI, with PsaA1 and PsaB1 being the major components under FR light, while PsaA3 and PsaB3 predominate under WL conditions. Under FR light PsaA2 and PsaB2 are minor components with spectral abundances less than 10% of the total PsaA/B pool, but are strongly differentially expressed under WL condition, with similar abundance patterns to PsaA3 and PsaB3 subunits for PSI isolated from WL (Additional file 2: Figure S9). The PSII core proteins D1 and D2, which are encoded by the *psbA* and *psbD* genes respectively, also account for an average ~ 1.0% of total protein and are of similar abundance to one another (Additional file 2: Figure S9). Abundance of the ChlF synthase, which is orthologous to *psbA* genes (See Additional file 2: Figure S2), increases 17-fold after one week under FR light compared with WL conditions, however this still only corresponds to 0.4% of the total D1 + D2 proteins under FR light (Fig. 7; Additional file 2: Fig. S9). However, based on our proteomic data we are unable to predict proteins that may be responsible for Chl *f* degradation that must be occurring, as Chl *f* decreased when cells are shifted from FR to WL light conditions [9, 24]. The modulation of the photosynthetic apparatus and ChlF synthase abundance highlights the specific structural and metabolic adaptations needed by Chl *f*-producing cyanobacteria to thrive under extreme light-limited conditions. The divergent phylogenetic group of the FR light-induced PsaA1 (Additional file 2: Fig. S1) suggests that common features of the protein environments in Chl *f*-producing cyanobacteria are required for binding pigments that differ from typical PsaA proteins in non-Chl *f*-producing cyanobacteria.

Heat shock proteins (HSPs) in bacteria, such as GroEL and DnaK, are commonly accumulated in response to environmental stresses [51]. All of these polypeptide-binding proteins are implicated in protein folding, protein targeting to membranes, renaturation, and in the control of protein-protein interactions, all of which are critical in supporting reconstruction of the photosynthetic apparatus in response to changed environments. Abundance of proteins involved in the maintenance of essential cellular processes such as DNA/RNA metabolic reactions is also independent of the switched light conditions (Additional file 3: Excel file S2).

CRISPR-Cas operons are commonly found in cyanobacteria isolated from biocommunity environments [44]. There are ten annotated repeat regions along with five annotated CRISPR-Cas operons in *H. hongdechloris* genome (Fig. 2).

Sequencing of the *H. hongdechloris* genome and analysis of the proteomic data revealed surprising details regarding its central carbohydrate metabolic pathways. Most cyanobacteria have an alternative TCA cycle, lacking a 2-oxoglutarate dehydrogenase, instead using two enzymes, 2-oxoglutarate decarboxylase and succinic semialdehyde dehydrogenase, to convert 2-oxoglutarate to succinate, thus forming the cyanobacterial complete TCA cycle shunt [52, 53]. We found that enzymes for converting 2-oxoglutarate to succinate were annotated in the *H. hongdechloris* genome (Additional file 2: Fig. S5). The proteomic data also support the activity of an ornithine bypass shunt which may provide additional fumarate for the TCA cycle (Additional file 2: Fig. S5; Additional file 3: Excel file S2). Additionally, the *H. hongdechloris* genomic information also confirmed the presence of a TCA cycle hypoxia shunt, allowing interconversions between pyruvate, oxaloacetate and malate (Additional file 2: Fig. S5). No genes encoding enzymes for cyanobacterial TCA cycle variants such as TCA glyoxylate or citramalate shunts [51] were found in the *H. hongdechloris* genome. The co-existing multiple TCA cycle shunts may play important roles in *H. hongdechloris* metabolic plasticity, balancing carbon and nitrogen assimilation under different conditions.

Our proteomic results support the role of the FaRLiP cluster in FR light adaptation in *H. hongdechloris* as has been observed for other characterized Chl *f*-producing cyanobacteria [15–17]. We detected an increase in the concentration of allophycocyanin subunits and increased levels of alternative subunits in photosystem reaction centres, including the core proteins for PSI and PSII under FR light (Fig. 4). Recently we defined P700 as the central chlorophyll in isolated PS I from cells grown under either WL or FR light conditions and that Chl *f* accounts for ~ 8% of total Chls from the isolated PSI from FR light-grown cells [25]. Nürnberg et al. demonstrated that Chl *f* acts as the primary donor in the position of Chl_D1_ in PSII and Chl_A-1_ in PSI, although the position of the other Chl *f* molecules [27] and their roles in the proposed up-hill energy transfers remain unclear [27–29].

The detailed global proteomic analysis reveals that more than 24% of the total mass spectral readings are photosynthetic proteins, affirming the importance of photosynthesis in cyanobacteria. The lack of changes in essential cellular metabolic reactions under shifted light conditions further support that changed light conditions mainly cause changes in photosynthetic subunits in order to maximize photosynthetic efficiencies under changed light environments.

## Methods

### Genomic DNA isolation

*Halomicronema hongdechloris* was grown and maintained in modified Seawater-KES medium as described in Li et al. 2014. Genomic DNA was prepared from approximately 200 mg wet weight of *H. hongdechloris.* The cells were washed with 10 mL of TE buffer (10 mM Tris-HCl (pH 8.0), 1 mM EDTA (pH 8.0)) and blotted dry in a mortar and pestle with filter paper. The cells were rinsed briefly with 70% ethanol and blotted dry again then frozen in liquid nitrogen and ground to a fine powder. The powder was added to 1 mL of CTAB buffer [100 mM Tris-HCl (pH 8.0), 20 mM EDTA (pH 8.0), 1.4 M NaCl, 2% (*w*/*v*) CTAB (cetyltrimethylammonium bromide), and 1% (w/v) PVP 40,000 containing 20 mg/mL Proteinase K and incubated at 65 °C for 10 min. To this, 1 mL of phenol:chloroform 1:1 *v*/v was added and mixed gently to form an emulsion. The emulsion was separated by centrifuging at 18,000 g for 10 min. The resulting upper layer containing genomic DNA was added to an equal volume of isopropanol and precipitated overnight at 4 °C. Genomic DNA was collected by centrifugation at 18,000 g for 20 min at 4 °C. The DNA was dissolved in TE buffer.

### Genome sequencing

Three different sequencing technologies were applied in order to get good genome coverage and to deal with confounding repetitive sequences. 1). For 454 GS FLX 25 Titanium sequencing genomic DNA was sheared and adaptors ligated according to the manufacturer’s instructions (Roche Diagnostics). Subsequently AMPure bead purification (New England BioLabs) was followed by emulsion PCR (emPCR) and sequenced on the Roche 454 Sequencing platform (Roche Diagnostics) at the Ramaciotti Centre for Gene Function Analysis (University of New South Wales Australia). 2). Genomic DNA was also sequenced (5000 bp mate paired-end library with 36 bp reads) on Solexa 1G at the Ramaciotti Centre for Gene Function Analysis (University of New South Wales Australia). 3). The genomic DNA was also sequenced using PacBio RS single molecule sequencing at the UC Davis sequencing centre (University of California Davis US). The errors in PacBio data were corrected using CABOG both in self correction mode and using the corrected and trimmed 454 reads following methods in Koren et al. 2012 [54]. An initial draft assembly consisted of 223 contigs (5313 nt to 299,419 nt in length N50 = 61,484, N90 = 17,660) based on the 454 and Solexa sequencing data. Analysis of these contigs with MUMmer [34] revealed that many of the smaller contigs were > 95% identical and/or had large > 10 kbp overlaps usually due to poor 454 reads at the end of the contigs. This was then confirmed in most cases by Sanger sequences of PCR products. The 454 and Illumina sequence reads were reassembled de novo in a hybrid assembly with MIRA 4.9 [33] with -CL:smrc = 100 for 454 data. This draft assembly consisted of 66 contigs greater than 5000 bp in length and had a total genome size of 5,900,685 nt with 54.6% GC content. The N50 was 383,857 nt and N90 was 148,650 nt and N95 25,199 nt was calculated to have an average coverage of 85× over all contigs with ~ 26 fold 454 and ~ 56 fold Solexa sequence coverage. Four of the contigs, making up ~ 200,000 bp had less than 8x Illumina coverage and zero 454 coverage and were discarded. An attempt was made to join the remaining 62 contigs in Gap5 [35] using the self-corrected PacBio reads. During this process, it appeared that there was a significant number of chimeric PacBio sequence reads. Use of 454 sequencing data to correct the PacBio reads reduced the number of chimeric reads. These remaining 62 contigs were then used in a mapping assembly with MIRA 4.9 with the 454 corrected PacBio reads. This assembly was merged with the assembly of the 62 contigs in Gap5 [35]. This strategy identified some small misassemblies on the ends of some contigs based on the PacBio data with subsequent large overlaps identified in 19 contigs, ranging from 2000 to 50,000 bp. Joins between the 19 contigs were confirmed by PCR and Sanger sequencing of the PCR products to yield a final scaffolded sequence of 5,574,084 with no gaps. Initially we were unable to close the genome due to poor sequence coverage near the ends of the final scaffold and a 350 bp region at the start which was repeated at ~ 1800 bp and at 3 other sites in the genome.

To close the genome 2000 bp of each end of this scaffold was blasted against all of the raw uncorrected PacBio reads identifying 38 reads. These reads were assembled with canu to yield two contigs [55]. One contig of ~ 4000 bp matched a region at ~ 640,000 bp in the genome and a second ~ 8000 bp spanned the two ends of the scaffold supplying an additional ~ 3700 bp of new sequence. In addition, the new ~ 3700 bp sequence was used as bait with mirabait to retrieve additional solexa and 454 sequences. All of these sequences were reassembled in a hybrid assembly with mira to obtain 3761 bp of sequence closing the genome. PCR primers were designed across this region, amplified and sequenced to correct the final sequence (Additional file 2: Figure S10). This spanning sequence contained two new coding sequences and proteomic evidence of expression was also obtained for the larger of these two coding sequences.

### Proteome analysis of *H. hongdechloris* in response to different light conditions

*H. hongdechloris* was grown for three weeks at 27 °C in modified KES medium under either Far-red light (~ 730 nm LED) illumination (FR-cells), or under fluorescent white light illumination (WL-cells) at a light intensity of 20 μmol photons m^− 2^ s^− 1^ [24]. FR-cells and WL-cells were equally divided into two subcultures and one was kept under the same illumination (FR-cells or WL-cells) and the other subcultures were switched to either WL or FR light conditions for 7 days: FR-cells to WL illumination (FRW-cells) or WL-cells to FR illumination (WR-cells). Due to the slow growth rate of *H.hongdechloris*, 7 days represents two doubling times, and corresponds to chlorophyll changes observed at this time, as previous reported [24]. The cells were harvested and washed in TE buffer twice. Approximately 100 mg of pelleted cells (wet weight) were resuspended in 1.5 ml pre-chilled 89.93% acetone, 10% trichloroacetic acid, and 0.07% β-mercaptoethanol to which protease inhibitors were added and incubated at − 20 °C for 45 min. Proteins were collected by centrifugation at 14,000 g at 4 °C for 20 min. The pellet was rinsed four times using pre-chilled 100% acetone and resuspended in 50 mM Tris-HCl buffer (pH 8.8) containing 2% (*w*/*v*) SDS. Insoluble material was precipitated by centrifugation at 14,000 g for 20 min at 4 °C and discarded. The solubilized total proteins were reduced using 5 mM DTT for 15 min at room temperature and alkylated using 10 mM iodoacetamide for 30 min in the dark at room temperature.

Alkylated protein samples were mixed with 4 volumes of 100% methanol and 1 volume of chloroform followed by 3 volumes of water. The samples were vortexed and incubated on ice for 5 min prior to centrifugation at 14,000 g for 5 min at 4 °C. After removing the organic solvent layer, total protein samples were washed using pre-chilled methanol, followed by pre-chilled acetone, then air dried. Dried total protein samples were resuspended in 50 mM Tris-HCl (pH 8.8) containing 8 M urea to a final concentration of at least 1 mg/ml as determined by Bradford assay. Tandem Mass Tag (TMT) proteome analysis was performed on the peptides obtained after Lys-C and Trypsin digestion and labelled in a 10-plex TMT reaction as per manufacturer’s instructions (ThermoFisher Scientific). The peptides were analysed using liquid chromatography electrospray ionization tandem mass spectrometry (Orbitrap, Thermo Scientific, US) and data collection using full MS scan (300˗1500 m/z) with automatic gain control (AGC) target of 5 × 10^5^ on Orbitrap followed by collision-induced dissociation (CID) analysis, with an AGC target of 4 × 10^3^ and 6 × 10^4^ for CID-MS2 and CID-MS3 analyses. The spectra were converted to mzxml format that were used to search against the *H. hongdechloris* predicted proteome and a reverse decoy database using X!TANDEM [56]. The search results were analysed using the TransProteomic Pipeline [57] and filtered to a ProteinProphet probability > 0.92 which corresponded to a protein false discovery rate of less than 0.5% based on hits in the reverse decoy database. TMT 10-plex relative quantification between different light treatments was performed on the MS2 fragment ion data using Libra. The relative quantification in each channel was scaled for small global differences in each of the labelled reporter ions such that global average pairwise comparisons between channels equalled 1.0. Relative protein expression compared to total protein in the cell was calculated using spectral counting normalized for total detected protein length (SAF) [58]. The relative abundance of individual proteins assumed the total spectral counts equalled 100% of the total protein.

### Phylogenetic analysis of genes of interest from *H. hongdechloris*

Predicted protein sequences derived from genes of interest from *H. hongdechloris*, including core reaction centre subunits of PS I (PsaA), PS II (PsbA) and light-harvesting chlorophyll-binding proteins (CP43’), were compared with sequences retrieved by a standard protein BLAST search against the database of reference proteins (refseq_protein) and downloaded. The sequences were aligned using ClustalW and the alignments were later refined manually according to conserved sites using MEGA 6 [59]. The neighbour joining (NJ) trees were constructed using the Dayhoff model and verified with 10,000 replicates in MEGA 6. Bootstrap values that supported a node in more than 50% of the replicate trees were retained.

### The effects of organic compounds on the growth of *H. hongdechloris*

*H. hongdechloris* was grown and maintained in modified Seawater-KES medium as described in Li et al. 2014 under fluorescent white light illumination at a light intensity of 20 μmol photons m^− 2^ s^− 1^, with stated organic compounds supplemented at either 0.1% or 1% (*w*/*v*) as indicated. The wet weight of cells at the sampling points was obtained by rinsing the cell pellets using Milli Q water and removing any remaining surface water by vacuum filtration before weighing. The growth curves presented are from three individual experiments with three technical replicates in each.

## Additional files

Additional file 1: **Excel file S1** List of insertion sequence (IS) elements and tandem repeats. (XLSX 100 kb)

Additional file 2: **Figure S1** Phylogenetic relationships of PsaA from cyanobacteria. **Figure S2.** Phyogenetic relationship of PsbA from cyanobacteria. **Figure S3.** Phylogenetic relationship of IsiA (CP43’) proteins from *H. hongdechloris* to counterparts from cyanobacteria. **Figure S4.** Carbon metabolic network. **Figure S5.** Tricarboxylic acid (TCA) cycle and alternative pathways annotated in the *H. hongdechloris* genome. **Figure S6.** Sugars and sugar derivatives metabolic pathways and *H. hongdechloris* growth rate was enhanced by polysaccharides supplement. **Figure S7.** The detected proteome protein function classification and distribution charts. **Figure S8.** Top 100 abundant protein (SAF greater than 0.2%) distributions. **Figure S9.** Peptide contributions to photosystem reaction centres. **Figure S10.** The insert DNA fragment closing the initial single linear sequence. A. Region of the chromosome joining the two ends of the initially linear contig. This region was especially difficult to map due to the presence of repeated sequences. To confirm that the chromosome was in fact circular we designed seven pairs of oligos covering this region (mapped on the chromosome using Artemis v13.2.0), and different regions of this fragment were amplified by PCR using different combinations of oligos (bidirectional blue arrows indicate the expected products). B. PCR products separated on a 0.8% agarose gel and stained with Sybr Safe (Invitrogen). To determine the molecular weight of the products we used the Bioline HyperLadder™ 1 kb (lanes MW). The lanes are numbered to coincide with the blue arrows in A. **Table S1.** List of genes encoding for photosynthetic pigment-binding protein complexes. **Table S2.** Gene list of Oxidative phosphorylation (PDF 2565 kb)

Additional file 3: **Excel file S2.** TMT proteomic data sets. (XLSX 497 kb)

## Abbreviations

ACP: allophycocyanin
Chl: chlorophyll
ChlH: protoporphyrin IX Mg-chelatase H subunit
ChlP: geranylgeranyl reductase
CRISPR: Clustered Regularly Interspaced Short Palindromic Repeats
ED: Entner-Doudoroff pathway
FaRLiP: far-red light photoacclimation
FR: far-red light
FR-cells: the cells grown under far-red light conditions
FRW-cells: the FR-cells switched to white light condition for 5–7 days
NSAF: normalised spectral abundance factor
OPP: Oxidative pentose phosphate pathway
PC: phycocyanin
PS: photosystem
SAF: Mass-based spectral abundance factor
TCA cycle: tricarboxylic acid cycle
TMT: Tandem Mass Tag
WL: white light. WL-cells, the cells grown under white light conditions
WR-cells: the WL-cells switched to far-red light conditions for 5–7 days
