# Supplementary material for: Genome and proteome of the chlorophyll f-producing cyanobacterium Halomicronema hongdechloris: adaptative proteomic shifts under different light conditions
Source: BMC Genomics. 2019 Mar 12;20:207. doi: 10.1186/s12864-019-5587-3 (PMC6416890; doi:10.1186/s12864-019-5587-3)
Supplement: Supplementary file 2 — Figure S1 Phylogenetic relationships of PsaA from cyanobacteria. Figure S2. Phyogenetic relationship of PsbA from cyanobacteria. Figure S3. Phylogenetic relationship of IsiA (CP43’) proteins from H. hongdechloris to counterparts from cyanobacteria. Figure S4. Carbon metabolic network. Figure S5. Tricarboxylic acid (TCA) cycle and alternative pathways annotated in the H. hongdechloris genome. Figure S6. Sugars and sugar derivatives metabolic pathways and H. hongdechloris growth rate was enhanced by polysaccharides supplement. Figure S7. The detected proteome protein function classification and distribution charts. Figure S8. Top 100 abundant protein (SAF greater than 0.2%) distributions. Figure S9. Peptide contributions to photosystem reaction centres. Figure S10. The insert DNA fragment closing the initial single linear sequence. A. Region of the chromosome joining the two ends of the initially linear contig. This region was especially difficult to map due to the presence of repeated sequences. To confirm that the chromosome was in fact circular we designed seven pairs of oligos covering this region (mapped on the chromosome using Artemis v13.2.0), and different regions of this fragment were amplified by PCR using different combinations of oligos (bidirectional blue arrows indicate the expected products). B. PCR products separated on a 0.8% agarose gel and stained with Sybr Safe (Invitrogen). To determine the molecular weight of the products we used the Bioline HyperLadder™ 1 kb (lanes MW). The lanes are numbered to coincide with the blue arrows in A. Table S1. List of genes encoding for photosynthetic pigment-binding protein complexes. Table S2. Gene list of Oxidative phosphorylation (PDF 2565 kb) [file 12864_2019_5587_MOESM2_ESM.pdf]

Additional Files' Content

Supplementary Figures

Supplementary Figure 1 (Figure S1). Phylogenetic relationships of PsaA from cyanobacteria.

Supplementary Figure 2 (Figure S2). Phylogenetic relationship of PsbA from cyanobacteria.

Supplementary Figure 3 (Figure S3). Phylogenetic relationship of isiA (CP43') proteins from *H. hongdechloris* to other cyanobacteria's counterparts.

Supplementary Figure 4 (Figure S4). Carbon metabolic network.

Supplementary Figure 5 (Figure S5). Tricarboxylic acid (TCA) cycle and alternative pathways annotated in the *H. hongdechloris* genome.

Supplementary Figure 6 (Figure S6). Sugars and sugar derivatives metabolic pathways and *H. hongdechloris* growth rate was enhanced by polysaccharides supplementary.

Supplementary Figure 7 (Figure S7). The detected proteome protein function classification and distribution charts.

Supplementary Figure 8 (Figure S8). Top 100 abundant protein (SAF greater than 0.2%) distributions.

Supplementary Figure 9 (Figure S9). Peptide contributions to photosystem reaction centres.

Supplementary Figure 10 (Figure S10). The insert DNA fragment closing the initial single linear sequence. A. Region of the chromosome joining the two ends of the initially linear contig. This region was especially difficult to map due to the presence of numerous repeated sequences. To confirm that the chromosome was in fact circular we designed seven pairs of oligos covering this region (mapped on the chromosome using Artemis v13.2.0), and different regions of this fragment were amplified by PCR using different combinations of oligos (bidirectional blue arrows indicate the expected products). B. PCR products separated on a 0.8% agarose gel and stained with Sybr Safe (Invitrogen). To determine the molecular weight of the products we used the Bioline HyperLadder™ 1kb (lanes MW). The lanes are numbered to coincide with the blue arrows in A.

Supplementary Tables

Supplementary Table 1. The List of genes encoded for photosynthetic pigment-binding protein complexes.

Supplementary Table 2. Gene list of Oxidative phosphorylation

Supplementary Excel files.

Supplementary Excel file 1. The List of Insertion Sequence (IS) elements and Tandem repeats.

Supplementary Excel file 2. TMT proteomic data sets.

Supplementary Figure S1

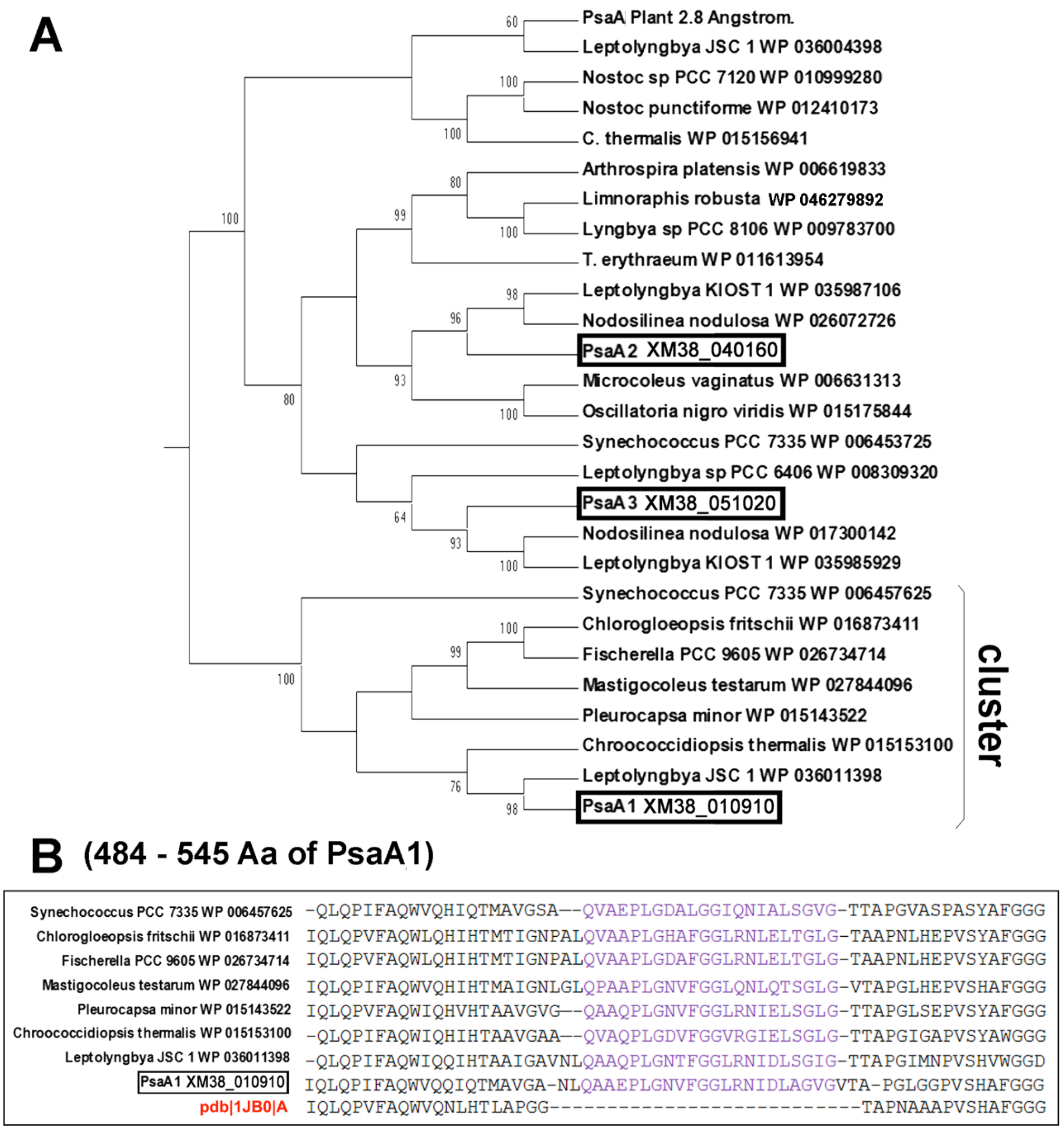

Figure S1. Phylogenetic relationships of PsaA from cyanobacteria. **A**. The neighbour-joining tree was constructed using the Dayhoff model in MEGA 6 with 10,000 replicates for verification. Bootstrap values (>50%) that support branches are presented. The sequence accession numbers are retained after cyanobacteria names. The three annotated PsaA's from the *H. hongdechloris* genome are XM38_010910, XM38_040160, and XM38_051020 (bold fonts in boxes). PsaA homologs from the known Chl *f*-producing cyanobacteria form a well supported clade. **B**, a special loop region at 484 – 545 amino acids (numbering from *H. hongdechloris*) from the special PsaA cluster was aligned and compared with the reference sequence pdb|1JB0|A from *Synechococcus elongatus*. The function of this addition loop awaits further experimental testing.

Supplementary Figure S2

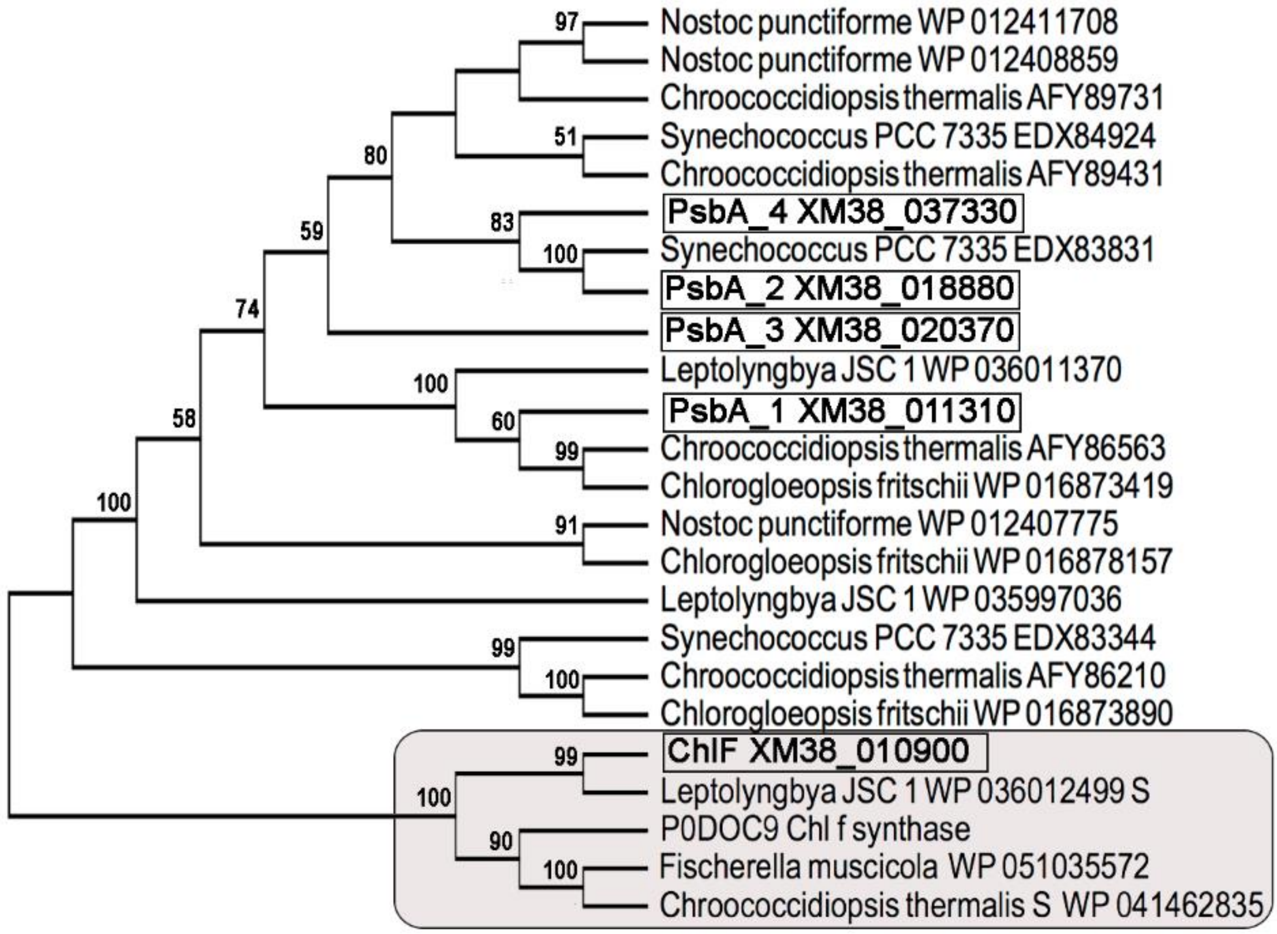

Figure S2. Phylogenetic relationship of PsbA from cyanobacteria. The five copies of PsbA homologs from the *H. hongdechloris* genome are in bold and boxed. The Neighbour-joining tree was constructed using the Dayhoff model in MEGA 6 and verified with 10,000 replicates. The bootstrape values (>50%) are presented on the branches. All sequence accession numbers are retained after cyanobacteria names. Shaded box, the ChlF homologs are grouped together with "P0DOC9 Chl *f* synthase" from *Chlorogloeopsis fritschii* PCC 9212 (Light-dependent Chl *f* synthase [24], also known as Super-rougue-PsbA4 [49]).

Supplementary Figure S3

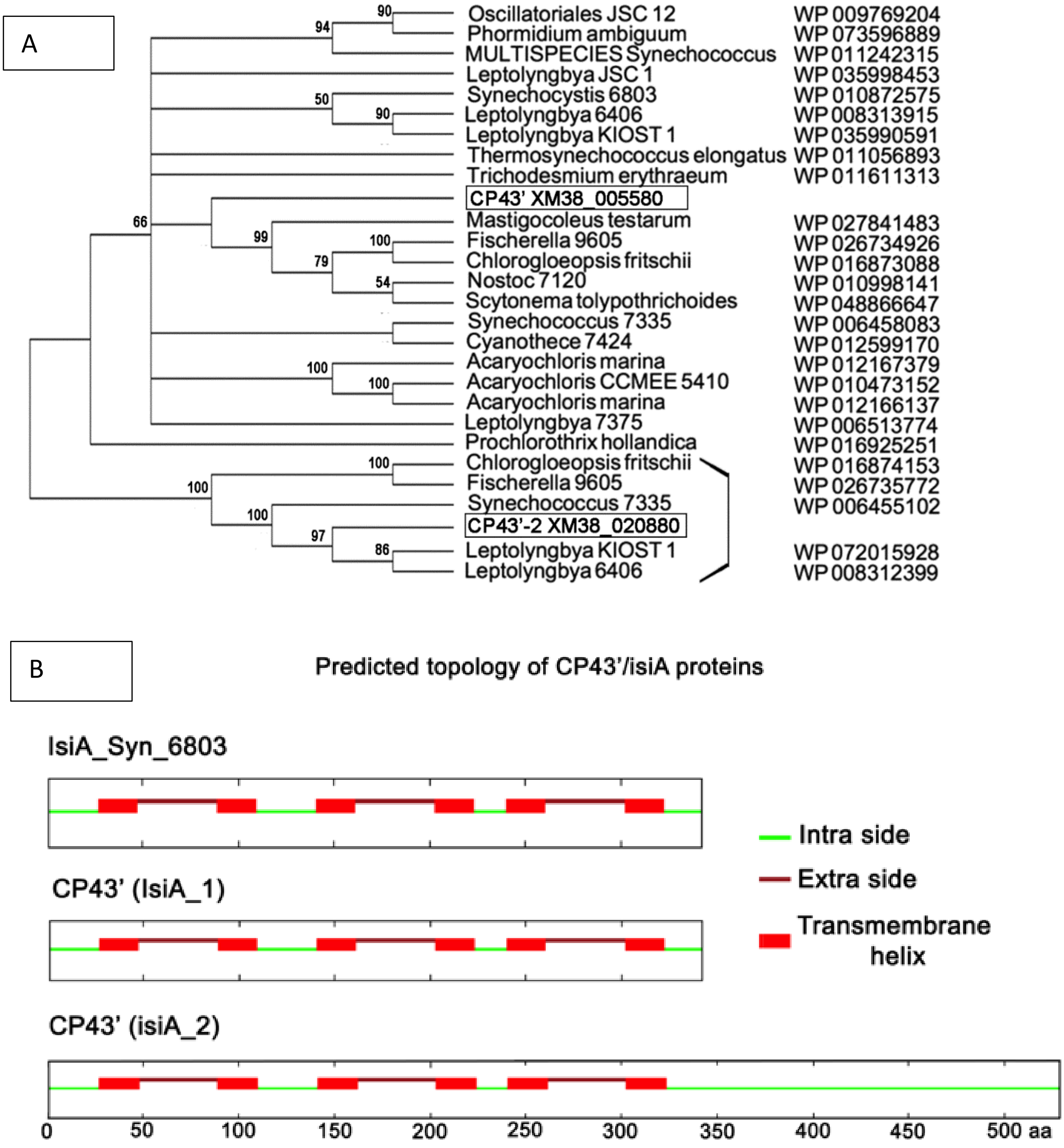

Figure S3. Phylogenetic relationship of isiA (CP43') proteins from *H. hongdechloris* to other cyanobacterial counterparts. **A**. Phylogenetic neighbor-joining (NJ) tree, which was constructed using the Dayhoff model in MEGA 6. The NJ tree was verified with 10,000 replicates and bootstrap values that supported a node of more than 50% of replicate trees are retained; **B**, predicted secondary amino acid structure of isiA proteins. Syn_6803 represents Synechocystis PCC 6803 (accession number: WP 010872575). CP43' (IsiA_1) is encoded by gene *XM38_005580* and CP43' (IsiA_2) is encoded by gene *XM38_020880* from *H. hongdechloris* genome. IsiA_2 from *H. hongdechloris* has an extra ~150 amino acid C-terminal tail that is predicted to extend out of the thylakoid.

Supplementary Figure S4

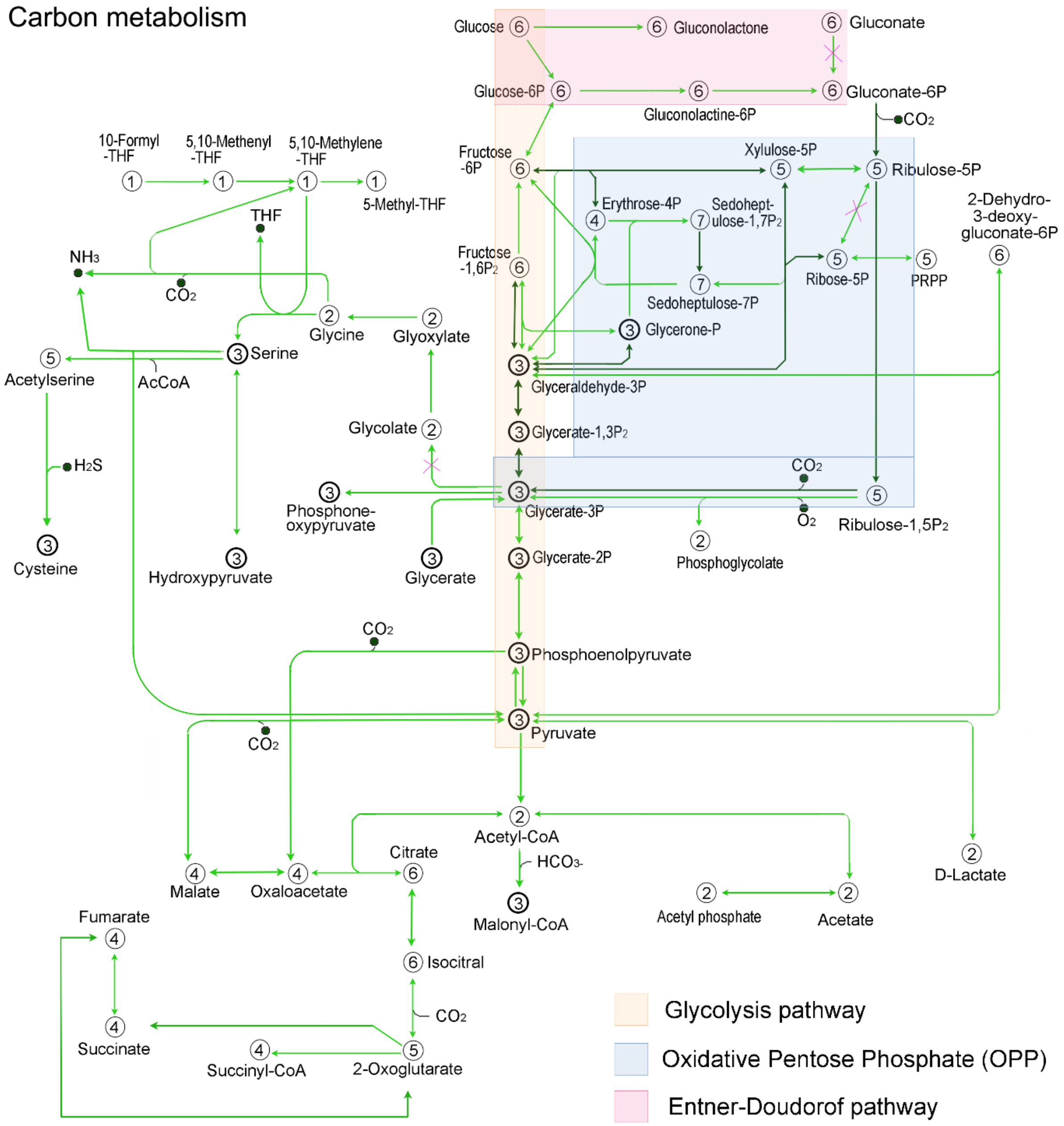

Figure S4. Carbon metabolic network. Numbers in the circles represent the number of carbon atoms in each substrate. The enzymes involved in the steps of Carbon fixation pathway are highlighted in solid dark green lines, which accounts for a total of >4.0% of proteins in *H. hongdechloris* based on total SAF. The three major carbohydrates metabolic pathway are highlighted using shaded colors. Red crosses over the reactions represent no annotated enzymes were found in the *H. hongdechloris* genome.

Supplementary Figure S5

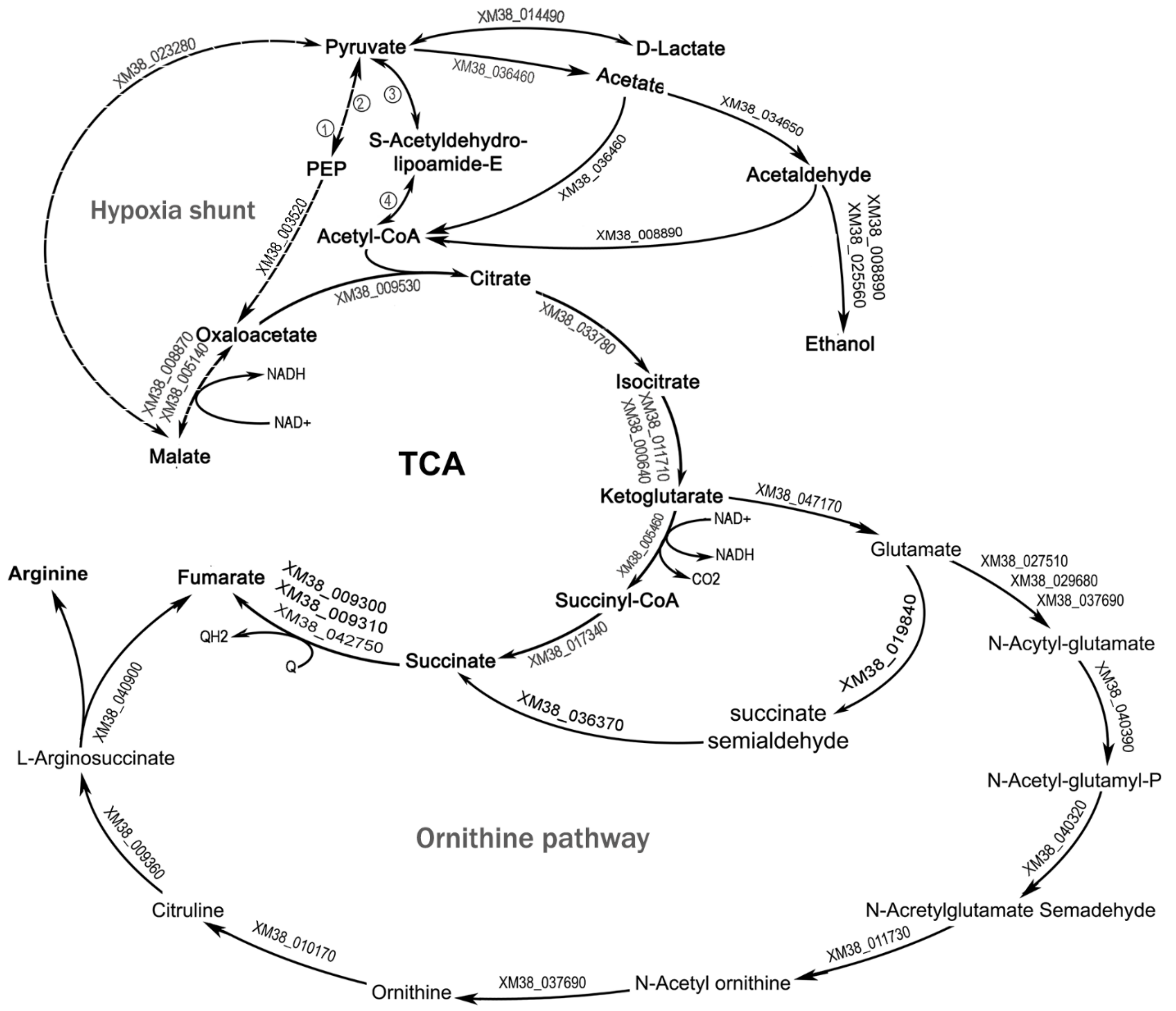

Figure S5. Tricarboxylic acid (TCA) cycle and alternative pathways annotated in the *H. hongdechloris* genome. The annotated enzymes are labelled using their gene identification number (XM38_0xxxx0). Multiple copies of genes encoding a number of enzymes in the pathway were identified. The dashed cycle represents the hypoxia shunt according to Steinhauser et al 2012 [52]. PEP, phosphoenolpyruvate. ① represents genes *XM38_008900* and *XM38_030260*; ② represents genes *XM38_010550*, *XM38_040150*, *XM38_042660*, and *XM38_052140*; ③ represents genes *XM38_033500* and *XM38_033510*; ④ represents genes *XM38_005460* and *XM38_004770*.

Supplementary Figure S6

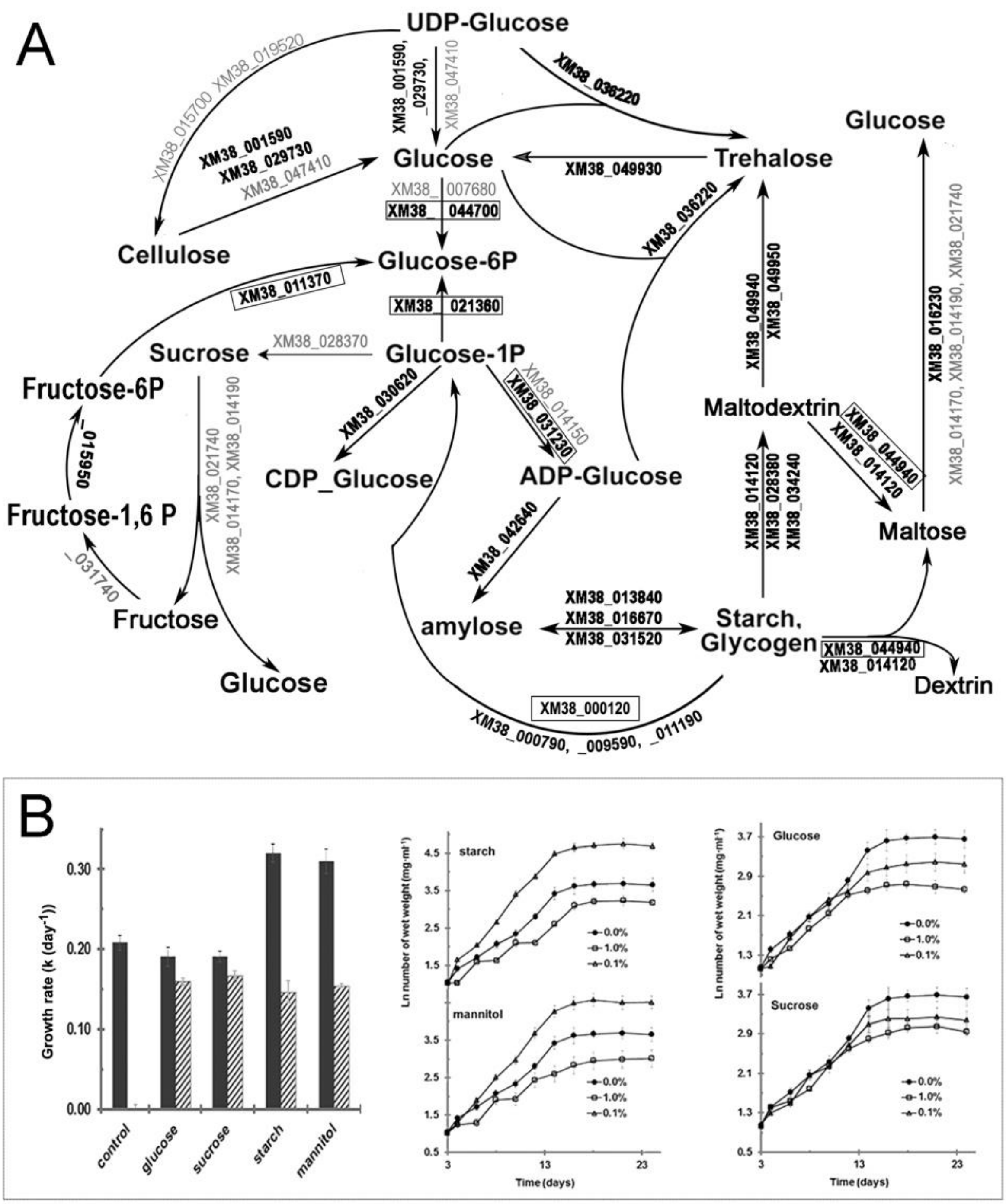

Figure S6. Sugars and sugar derivatives metabolic pathways and *H. hongdechloris* growth response to supplementary polysaccharides. **A**, Sucrose and starch metabolic pathway. Gene ID's of those annotated to encode particular enzymes in the pathway are labelled. Relatively abundant enzymes, having SAF >0.2%, are in bold and in boxes. Enzymes with an SAF <0.2 and >0.1% are in boxes. The enzymes in bold represent those proteins with an SAF <0.1% and >0.001%. The grey colored enzymes were undetectable in our proteomic analysis (<0.001% SAF). **B**, The effects of organic compounds on the growth of *H. hongdechloris* culture. *H. hongdechloris* growth was enhanced by supplementary starch (0.1%) and mannitol (0.1%), which agrees well with the presence of annotated metabolic pathways (Fig. S6A). The black bars in Figure 6B represent the concentration of supplemented organic compounds in 0.1% (w/v); the striped bars represent the concentration of supplemented organic compounds in 1.0% (w/v). k represents the growth rate constant, expressed in 6B as reciprocal days (day^{-1}). Error bars represent standard error (n=8).

Supplementary Figure S7

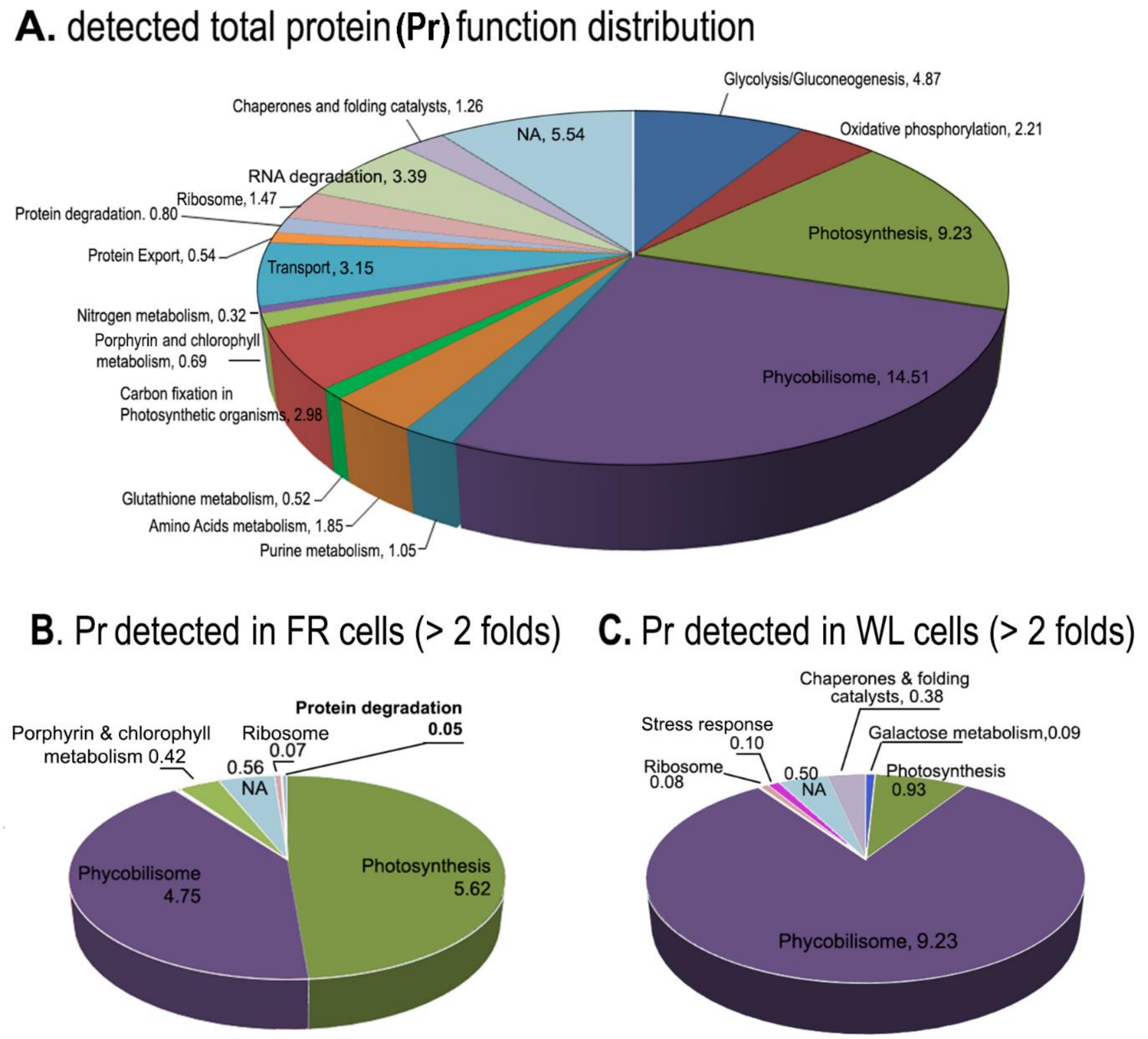

Figure S7. Functional classification and distribution of proteins detected in the *H. hongdechloris* proteome. **A**, Total protein function distribution charts of detected proteins (SAF >0.001% of total SAF). **B**, Functional category distribution of proteins with >two-fold abundance in far-red (FR) light grown cells compared to white light (WL) cells. **C**, Functional category distribution of proteins with >two-fold abundance in WL grown cells compared to FR light cells.

Supplementary Figure S8

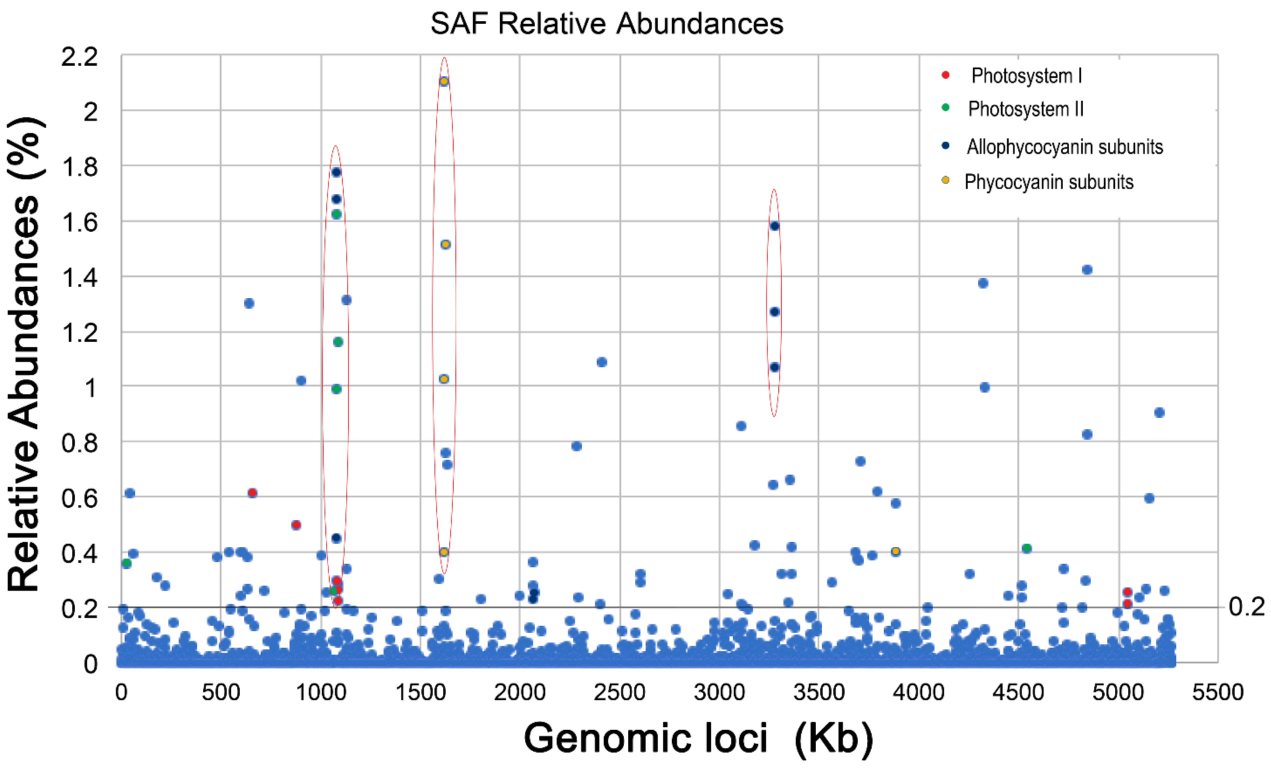

Figure S8. Top 100 most abundant proteins (SAF greater than 0.2%) in the *H. hongdechloris* proteome. The oval shapes highlight clusters of highly abundant proteins. Red dots, photosystem II subunits, green dots, photosystem II subunits; dark blue dots, allophycocyanin subunits and orange dots are phycocyanin subunits.

Supplementary Figure S9

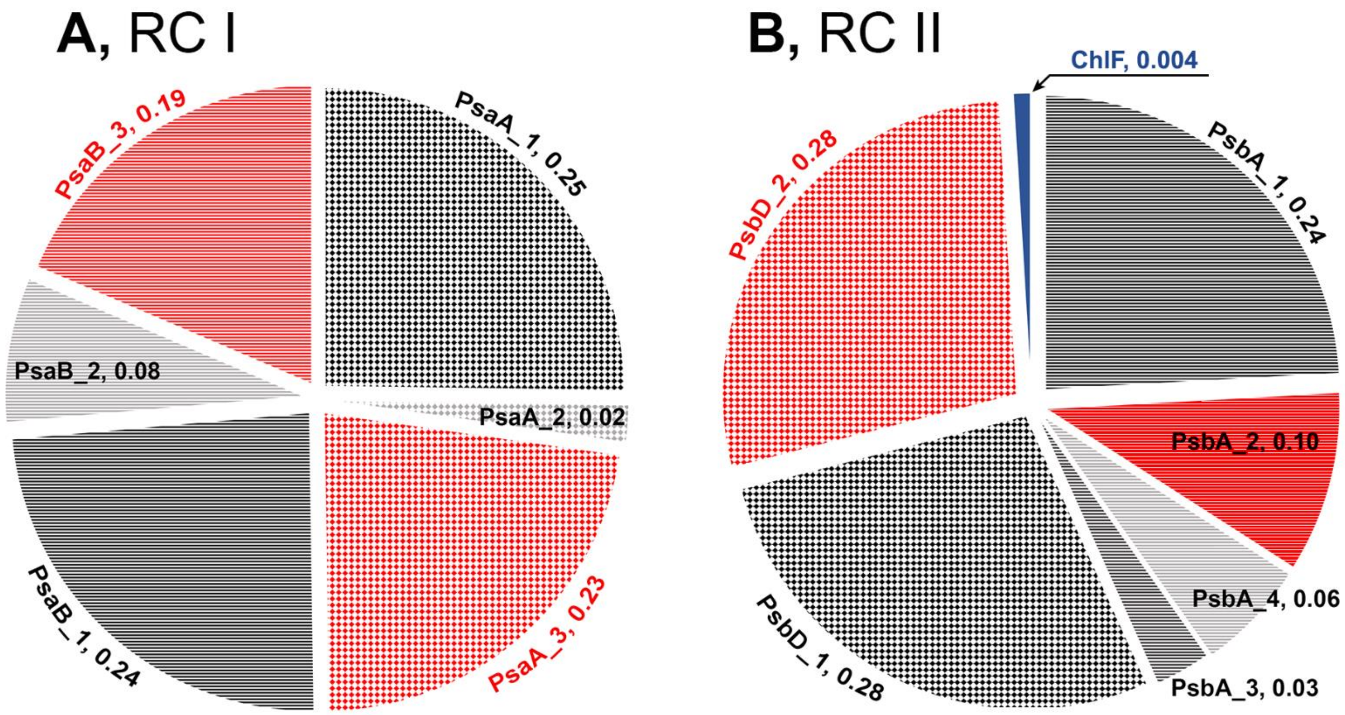

Figure S9. Peptide contributions to photosystem reaction centres. **A**, Photosystem I reaction centre (RCI) peptides distributions. Cross-hatch and striped areas represent PsaA and PsaB peptides respectively. The numbers after protein names represent SAF reading percentages. Total SAF (%) for PsaA and PsaB peptides are approximately equal. Red colour represents peptides stimulated under FR light conditions; **B**, Photosystem II reaction centre (RCII) peptide distributions. Striped and cross-hatch areas represent PsbA (D1) and PsbD (D2) peptides respectively. The numbers after protein names represent SAF reading percentages. Red colour represents stimulated peptides under FR light conditions. Chlf synthase (ChlF), which is strongly stimulated under FR light, is marked in blue (SAF = 0.004). SAF is the percentage of detected target peptide length per the sum of total detected peptide length (%).

Supplementary Figure S10

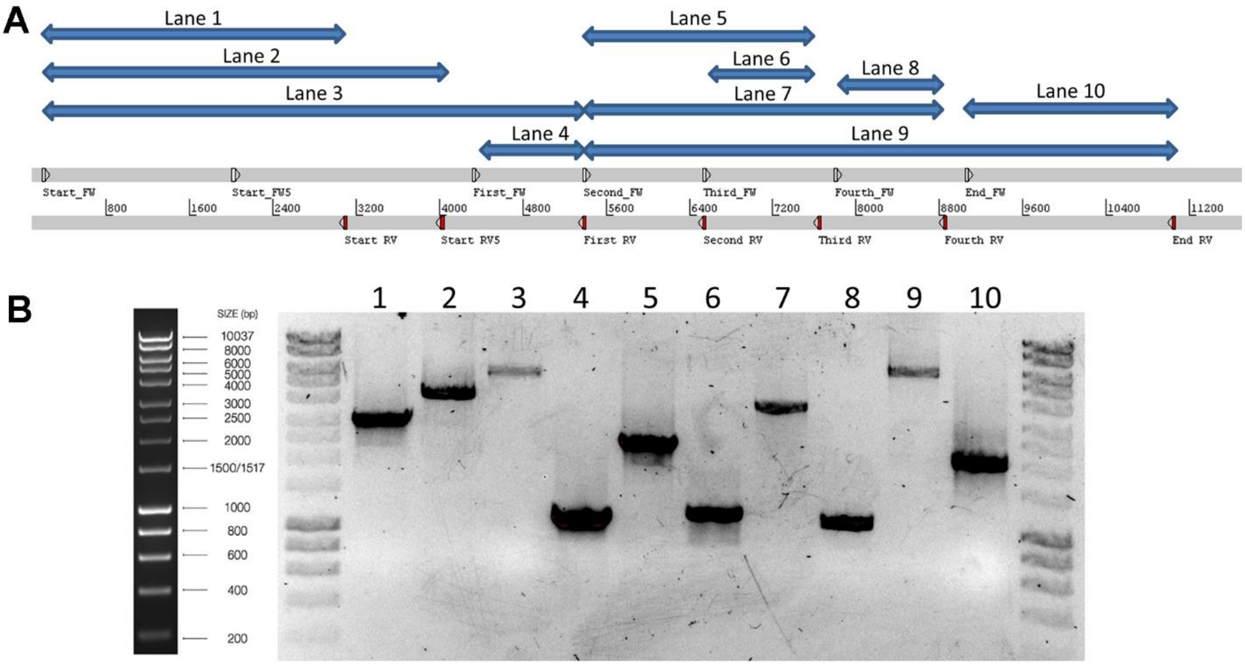

Figure S10. The insert DNA fragment closing the initial single linear sequence. A. Region of the chromosome joining the two ends of the initially linear contig. This region was especially difficult to map due to the presence of repeated sequences. To confirm that the chromosome was in fact circular we designed seven pairs of oligos covering this region (mapped on the chromosome using Artemis v13.2.0), and different regions of this fragment were amplified by PCR using different combinations of oligos (bidirectional blue arrows indicate the expected products). B. PCR products separated on a 0.8% agarose gel and stained with Sybr Safe (Invitrogen). To determine the molecular weight of the products we used the Bioline HyperLadder™ 1kb (lanes MW). The lanes are numbered to coincide with the blue arrows in A.

Supplementary Tables

Table S1 List of genes encoding peptides for photosynthetic pigment-binding protein complexes

A	Names	Gene ID	B	Names	Gene ID	C	Names	Gene ID
Photosystem I (Psa)#	*psaA1*	XM38_010910	Photosystem II (Psb)#	*psbA1*	XM38_010770	Allophycocyanin (Apc)	*apcA*	XM38_033080
	psaB1	XM38_010920		*psbA2*	XM38_018880		*apcB1*	XM38_010860
	psaA2	XM38_040160		*psbA3*	XM38_020870		*apcB2*	XM38_020890
	psaB2	XM38_040170		*psbA4*	XM38_037330		*apcB3*	XM38_033090
	psaA3	XM38_051020		*psbB1*	XM38_006080		*apcB4*	XM38_037370
	psaB3	XM38_051010		*psbB2*	XM38_010800		*apcC*	XM38_033100
	psaC	XM38_031380		*psbC1*	XM38_010810		*apcD1*	XM38_010830
	psaD	XM38_006600		*psbC2*	XM38_045920		*apcD2*	XM38_010850
	psaE	XM38_008840		*psbD1*	XM38_010820		*apcD3*	XM38_010870
	psaF1	XM38_010950		*psbD2*	XM38_016040		*apcD4*	XM38_020900
	psaF2	XM38_047750		*psbD3*	XM38_045910		*apcD5*	XM38_031490
	psaI1	XM38_002300		*psbE*	XM38_031940		*apcE1*	XM38_010840
	psaI2	XM38_010940		*psbF*	XM38_031950		*apcE2*	XM38_033070
	psaI3	XM38_040190		*psbH1*	XM38_010780	Phycocyanin (Cpc)	*cpcA*	XM38_016410
	psaJ2	XM38_047740		*psbH2*	XM38_015860		*cpcB*	XM38_016420
	psaJ1	XM38_010960		*psbI*	XM38_016110		*cpcC1*	XM38_016390
	psaK	XM38_037430		*psbJ*	XM38_031960		*cpcC2*	XM38_016400
	psaL1	XM38_010930		*psbK*	XM38_000470		*cpcD*	XM38_016380
	psaL2	XM38_040180		*psbM*	XM38_006060		*cpcE*	XM38_016370
	psaL3	XM38_047730		*psbN**	XM38_015850		*cpcF*	XM38_016360
				psbO1	XM38_000220		*cpcG*	XM38_039210
				psbO2	XM38_010880		Chl-binding proteins	
				psbP	XM38_015750		*isiA1*	XM38_005580
				psbQ	XM38_038660		*isiA2*	XM38_020880
				psbU1	XM38_002180			
				psbU2	XM38_007430			
				psbV1	XM38_010290			
				psbV2	XM38_010890			
				psbX	XM38_025940			
				psbY	XM38_044580			
				psbZ	XM38_052060			
				*psb27**	XM38_052960			
				ChlF	XM38_010900			

Red fonts represent the photosynthetic genes within the FaRLiP (far-red light photoacclimation) gene cluster.

*PsbN and Psb27 are assembly factor, not functional PSII subunit.

there are missing subunits from the genome, including psaM, psbL, psb28, psb29 and psbT.

Table S2 List of genes predicted to encode enzymes involved in oxidative phosphorylation in *H. hongdechloris*

	gene names	gene id
NADH dehydrogenase (Complex I)	*ndhA*	XM38_009540
	ndhC	XM38_031910
	ndhD1	XM38_016450
	ndhD2	XM38_045110
	ndhD3	XM38_050760
	ndhE	XM38_009570
	ndhF1	XM38_016460
	ndhF2	XM38_045100
	ndhF3	XM38_050750
	ndhG	XM38_009560
	ndhH	XM38_031730
	ndhI	XM38_009550
	ndhJ	XM38_031890
	ndhK	XM38_031900
	ndhL	XM38_030000
	ndhM	XM38_041540
	ndhN1 (ndhB)	XM38_035260
	ndhO	XM38_008860
	ndhN2	XM38_052930
Succinate dehydroge nase	*sdhA*	XM38_009300
	sdhB	XM38_042750
	sdhC	XM38_009310
Cyt C oxidas e	*cydA*	XM38_051950
	cydB	XM38_051940
ATPase (Complex V)	*atpA (alpha)*	XM38_032980
	atpB1 (a)	XM38_033030
	atpC (epsilon)	XM38_043610
	atpD (beta)	XM38_043620
	atpF1 (b)	XM38_033000
	atpF2 (b)	XM38_033010
	atpG (gamma)	XM38_032970
	atpH1 (delta)	XM38_032990
	atpH2 (c)	XM38_033020
	atpI	XM38_033040
